# Ultrahigh Seebeck Coefficient and Power Factor in Low‐Temperature Fused Ag_2_Se Films via Superionic‐Driven Plastic Deformation

**DOI:** 10.1002/advs.202508381

**Published:** 2025-09-16

**Authors:** Dezhuang Ji, Baosong Li, Xuan Li, Husam Hashem AlTakrori, Balamurugan Thirumalraj, Moh'd Rezeq, Wesley Cantwell, Lianxi Zheng

**Affiliations:** ^1^ Department of Mechanical and Nuclear Engineering Khalifa University of Science and Technology P.O. Box Abu Dhabi 127788 UAE; ^2^ Department of Aerospace Engineering Khalifa University of Science and Technology P.O. Box Abu Dhabi 127788 UAE; ^3^ Research & Innovation Center for Graphene and 2D Materials (RIC‐2D) Khalifa University of Science and Technology P.O. Box Abu Dhabi 127788 UAE; ^4^ Research and Innovation on CO2 and H2 Center (RICH) Khalifa University of Science and Technology P.O. Box Abu Dhabi 127788 UAE; ^5^ Department of Physics Khalifa University of Science and Technology P.O. Box Abu Dhabi 127788 UAE

**Keywords:** Ag_2_Se, energy filtering, grain boundary, plastic deformation, superionic, Thermoelectric

## Abstract

Thermoelectric technologies enable the direct solid‐state conversion of heat into electricity, but performance improvement is challenging due to the strong interdependences among parameters. Here, the grain boundary‐based energy filtering effect is explored to selectively scatter the low‐energy carriers and thus enhance the Seebeck coefficient without degrading other properties. A superionic‐induced fluid‐like plastic deformation mechanism is utilized to fabricate dense and flexible Ag_2_Se films at a very low temperature (≈150 °C), which not only effectively sinters nanoparticles, but is also sufficiently low to maintain a high density of effective grain boundaries by suppressing excess fusing. The success of this low‐temperature fusion process is attributed to the existence of excess Ag atoms, which serve as fusing agents to enhance the plastic deformation and thus facilitate the formation of closely contacted grains. As a result, the grain boundaries between those close‐contacted grains boost the energy filtering effect while maintain the high electric conductivity, and therefore the obtained Ag_2_Se films achieve an outstanding Seebeck coefficient of −215 µV K^−1^ and a high power factor of 2500 µW m^−1^ K^−2^ at room temperature, representing a significant advancement in room temperature thermoelectric materials.

## Graphical abstract

Driven by superionic‐induced plastic deformation, low‐temperature treatment can not only effectively fuse Ag_2_Se nanoparticles into a dense film but also enhance thermoelectric performance by maximally preserving effective grain boundaries resulting in intensive energy filtering effect. Notably, excess Ag nanoclusters act as fusing agents, promoting the ‘sintering’ process of nanoparticles.

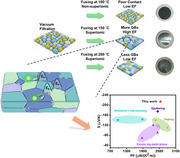

## 1 Introduction

Thermoelectric (TE) technologies enable the direct conversion of heat into electricity in a solid‐state manner, offering a viable approach for harvesting low temperature waste heat. Tremendous effort on performance enhancement has been paid via a variety of strategies, including multiscale microstructures from alloying,^[^ ^1^ ^]^ optimizing carrier density by doping,^[^ ^2^, ^3^ ^]^ and implementing band engineering such as band alignment^[^ ^4^, ^5^ ^]^ and resonant states^[^ ^6^ ^]^ to reshape the density of states (DOS).^[^ ^7^ ^]^ Unfortunately, most of these methods either fundamentally alter the intrinsic property/structure of the TE materials due to alloying and heavy doping,^[^ ^8^ ^]^ or improve one performance parameter at the expense of another due to the strong inter‐dependences among Seebeck coefficient, power factor, and thermal conductivity.

In comparison, energy filtering (EF)^[^ ^9^, ^10^ ^]^ method, an approach utilizing energy‐dependent scattering centers to selectively filter low‐energy carriers, offers a more promising alternative to enhance the Seebeck coefficient without compromising the power factor and thermal conductivity, as well as preserving other intrinsic properties and structures of the TE materials,^[^ ^9^ ^]^ but it is much less studied due to the difficulty in realizing and controlling the energy barriers.

The impacts of energy filtering effect can be explained by the following expressions derived from Boltzmann transport Equations (1) and (2):^[^ ^11^ ^]^

(1) $$\sigma \left(E\right)=\nu^{2}\;\tau \frac{-\partial f}{\partial E}D\left(E\right)$$

(2) $$S=\frac{{\langle \frac{E-E_{F}}{T}\rangle }_{\sigma }}{-q}$$

Where *σ*(*E*) is energy‐dependent electrical conductivity, *E* is the energy of charger carriers, *ν* is velocity of charger carriers, *τ* is the scattering time, *f* is Fermi‐Dirac distribution function, *D*(*E*) is energy dependent DOS, *S* refers to the Seebeck coefficient, *E_F_* refers to Fermi energy, *T* is temperature and *q* refers to charge.

**Figure 1:**
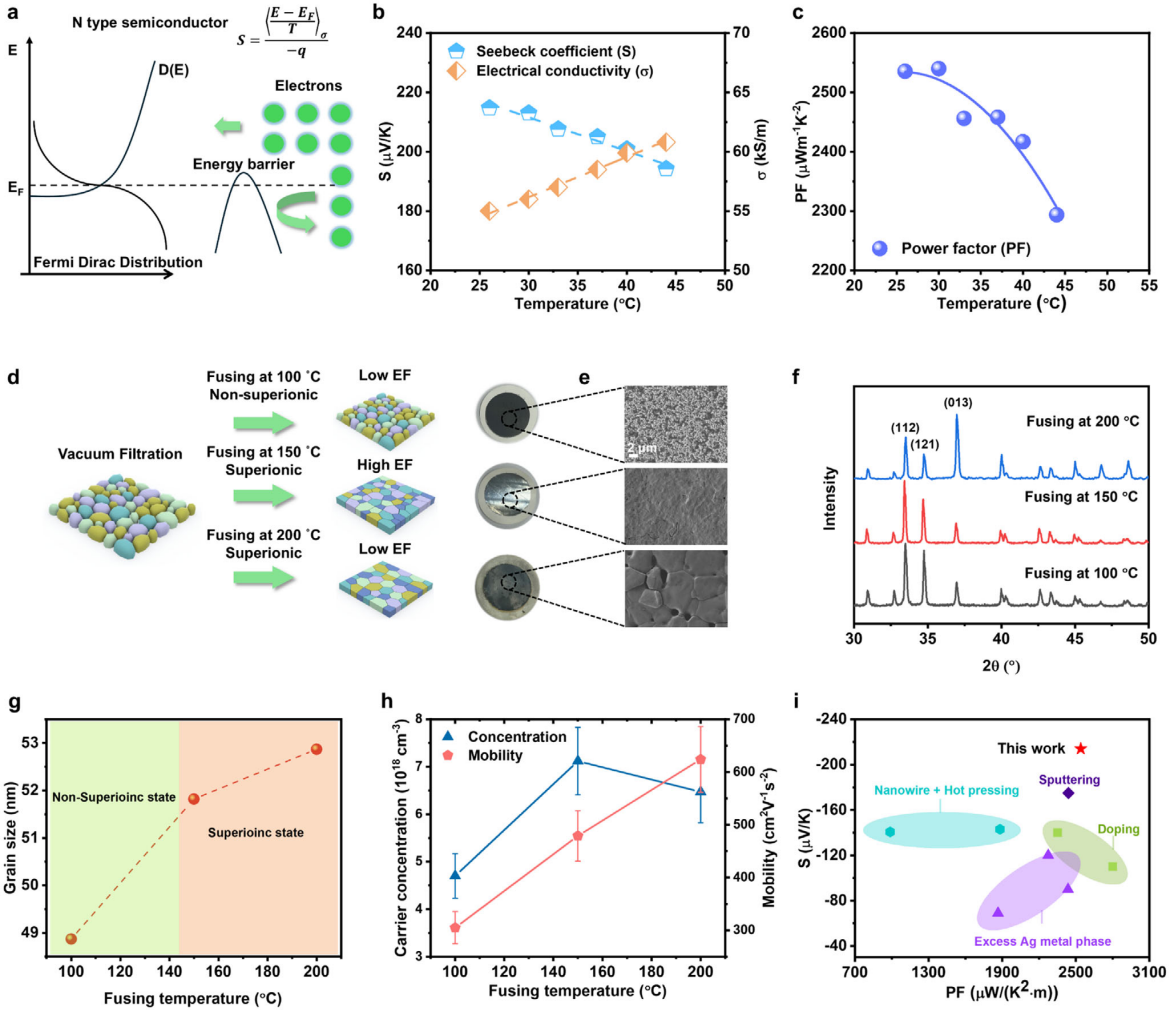
High TE performance in Ag_2_Se films induced by a strong EF on GBs. a) Schematics of energy filtering effect. b) Seebeck coefficient and electrical conductivity, as well as (c) power factor with respect to temperatures (Ag_2_Se film fused at 150 °C). d) Illustration of the film fusing process and the temperature influence on the formation of GBs and EF. e) SEM images of Ag_2_Se films fused at different temperatures. f) X‐ray diffraction (XRD) pattern and (g) corresponding grain size calculated using Scherrer equation of the Ag_2_Se films fused at 100, 150 and 200 °C. h) The charge concentration and mobility of Ag_2_Se films with respect to the fusing temperature. i) Comparison of Seebeck coefficient and power factor of Ag_2_Se films.

These expressions indicate that higher‐energy electrons contribute more significantly to the *S* than lower‐energy electrons, as *S* can be interpreted as the conductivity‐weighted average energy of charge carriers, and electrons with energies below the Fermi level contribute to *S* with the opposite sign compared to those above the Fermi level. Consequently, if low‐energy electrons, including those below the Fermi level, are effectively scattered and prevented from participating in transport, the conductivity‐weighted average energy and thus the *S* will be significantly enhanced (**Figure** **1**a).

Secondary phases are usually introduced to trigger energy filtering effects. For instance, Ko et al.^[^ ^12^ ^]^ reported an enhanced Seebeck coefficient in p‐type Sb_2_Te_3_ containing Pt nanocrystals, and Zhang et al.^[^ ^13^ ^]^ developed a strategy for the stable embedding of Ag nanoparticles in p‐type Sb_2_Te_3_. A theoretical model was proposed by Faleev and Léonard^[^ ^14^ ^]^ to explain the TE performance enhancement induced by metallic inclusions in n‐type PbTe, which suggested that the energy barriers at the inclusion‐matrix interface preferentially scatter low‐energy electrons while allow high‐energy electrons to pass with minimal impact. Beyond metallic nano‐inclusions, semiconducting inclusions have also been utilized to improve TE performance via energy filtering effect. Notable examples include Sb_2_O_3_ embedded in p‐type Bi_0.5_Sb_1.5_Te_3_,^[^ ^15^ ^]^ BiCuSeO@SnO_2_ core–shell nanostructures embedded in p‐type SnTe,^[^ ^16^ ^]^ and Ag_2_Te nanoprecipitates in PbTe.^[^ ^17^ ^]^ Magnetic materials have also been demonstrated to introduce additional energy barriers that selectively scatter low‐energy electrons, further enhancing TE performance.^[^ ^18^ ^]^ However, introducing a secondary phase is not always risk‐free. Its presence state is largely dictated by the synthesis process and can be difficult to control. Furthermore, incorporating secondary phase sometimes causes doping, which may trigger multiple effects, making the overall impact on TE performance limited and unpredictable.

On the other hand, grain boundaries (GBs) predominately exist in all polycrystalline TE materials, and they are electrically active due to charge trapping^[^ ^9^, ^10^ ^]^ at localized states that arise from impurities, dislocations, interfacial defects, or structural variations at the GBs. The energy filtering effect on electron transport is primarily governed by both the density and the strength of these energy barriers on GBs.^[^ ^9^ ^]^ The density of energy barriers can be straightforwardly tuned by controlling the amount of GBs. In contrast, modifying the height of energy barriers is more complex and depends on specific cases. For example, it may depend on impurity atoms at GBs, particularly in materials synthesized by wet chemical methods. Additionally, the degree of atomic crystallization at GBs also influences the height of energy barriers.

Although the formation of energy barriers at GBs and their potential to the energy filtering effect were studied theoretically,^[^ ^9^ ^]^ experimental study is very limited and energy filtering effect was only observed in the materials processed at low sintering temperatures,^[^ ^19^ ^]^ primarily due to the reduction in energy barrier caused by high‐temperature homogenization and the decrease in GBs density resulting from grain growth.

Recently, Ag_2_Se has gained significant attention because it exhibits similar TE performance but is more mechanical robust,^[^ ^20^ ^]^ compared to the only commercialized room temperature TE material Bi_2_Te_3_.^[^ ^21^ ^]^ A high *zT* of 1.2 at room temperature was reported with strategies of microstructural tailoring.^[^ ^22^, ^23^, ^24^ ^]^ In addition, Chen's group boosted Ag_2_Se films to a device level with a power factor of over 2900 µW m^−1^ K^−2^ observed at 393 K,^[^ ^25^ ^]^ and further to a value of 3080 µW m^−1^ K^−2^ at 343 K in their another study^[^ ^26^ ^]^ using orientation control strategy. Ag_2_Se is a phase change material, and it is superionic in its high‐temperature phase, with Ag ions exhibiting high ionic mobility within the rigid Se sublattice, resulting in significant phonon scattering and consequently low thermal conductivity. Meanwhile, the rigid Se sublattice governs the band structure, ensuring the preservation of high electrical conductivity, making it as a representative member of the novel class of TE materials, known as phonon‐liquid electron‐crystal (PLEC)^[^ ^27^ ^]^ materials including Ag_2_(S, Se, Te)^[^ ^22^, ^28^, ^29^ ^]^ and Cu_2_(S, Se, Te).^[^ ^27^, ^30^, ^31^ ^]^ And most importantly, the weak bonding between chalcogen and transition metal atoms in this group of superionic compounds endows them metal‐like plasticity, with an elongation of up to 4.2% and compressive and bending deformations exceeding 50% at room temperature being observed in Ag₂S.^[^ ^32^ ^]^ It is expected that such a superionic‐induced fluid‐like plastic deformation^[^ ^33^, ^34^, ^35^, ^36^ ^]^ allows the effective “sintering” of TE materials at a very low‐temperature^[^ ^37^ ^]^ to suppresses the growth of nanoparticles, which is greatly beneficial in preserving or maximizing the GBs between closely‐contacted grains for energy filtering study, compared to conventional high‐temperature sintering techniques such as spark plasma sintering (SPS)^[^ ^38^, ^39^, ^40^ ^]^ and hot pressing (HP).^[^ ^41^, ^42^ ^]^

Therefore, in this study, a low‐temperature fusing strategy based on superionic‐induced fluid‐like plastic deformation is employed to prepare dense and flexible Ag_2_Se films at a temperature slightly above its phase transition point (≈406 K), aiming at increasing the concentration of GBs with effective energy barrier to enhance the energy filtering effect. The obtained Ag_2_Se film achieves simultaneous enhancements in both the Seebeck coefficient (‐215 µV k^−1^) and electrical conductivity, yielding a power factor of ≈2500 µW m^−1^ K^−2^ at room temperature. The detail enhancement mechanism via GBs, as well as the presence and the role of excess Ag in accelerating the plastic deformation are extensively studied.

## 2 Results

In this research, the Ag_2_Se films were prepared by synthesizing Ag_2_Se nanoparticles followed by sintering or fusing at a very low temperature to create GBs with energy barriers. Ag_2_Se is a phase transition material with an orthorhombic structure (β‐Ag_2_Se) below 406 K and a cubic structure (α‐Ag_2_Se) above this temperature. This phase transition is reversible with temperature.^[^ ^43^ ^]^ Notably, the cubic phase exhibits superionic behavior, characterized by mobile Ag ions within the Se sublattice. Therefore, such weakened bonding between Ag and Se enables fluid‐like plastic deformation,^[^ ^33^ ^]^ and allows us sintering Ag_2_Se nanoparticles at a low temperature.^[^ ^44^ ^]^ In general, fluid‐like deformation allow the nanoparticles to reshape and fill the voids and pores, forming a dense film, while grain‐to‐grain bonding is further enhanced due to the ease of merging cubic structure^[^ ^43^ ^]^ (isotropic atomic spacing along all three crystallographic directions) as well as the exchange of mobile Ag ions within the Se sublattice across GBs.^[^ ^33^ ^]^

Inspired by the 2‐step methods of synthesizing Ag_2_Se nanoparticles or nanowires,^[^ ^45^, ^46^, ^47^ ^]^ which are beneficial in particle size control, a simplified 2‐step method was employed to synthesize Ag_2_Se nanoparticles.^[^ ^48^ ^]^ In this study, Ag_2_Se nanoparticles were synthesized at room temperature. The Se nanoparticles were synthesized via a wet chemical method, exhibiting an average size of 150 nm, as shown in the scanning electron microscopy (SEM) image (Figure S1a, Supporting Information). They then reacted with AgNO_3_ in a mild reducing agent, ethanol glycol (EG), to produce Ag_2_Se nanoparticles, which show a diameter of ≈ 150 nm (Figure S1b, Supporting Information), demonstrating a mild reaction manner without leading to significant growth of grains.^[^ ^46^ ^]^ The as prepared Ag_2_Se nanoparticles were then dispersed in ethanol assisted with sonication and filtrated on a nylon membrane to form a porous Ag_2_Se film, which was further fused at 150 °C for 1 h under the pressure of 1 MPa. The effectiveness of the low‐temperature fusing process is confirmed by a smooth surface morphology, with a roughness below 0.2 µm (excluding pore regions), as characterized by atomic force microscopy (AFM) (Figure S2, Supporting Information). The film exhibits a thickness of ≈10 µm (Figure S3a, Supporting Information), and its large‐area uniformity is validated by profilometer measurements (Figure S3b, Supporting Information). The discrepancy between the SEM and profilometer measurements may arise from non‐uniform compression of the Nylon film, as shown in the inset of Figure S3b (Supporting Information). The SEM measurement is considered more reliable in this context.

Such obtained Ag_2_Se films are found to exhibit a high Seebeck coefficient ‐215 µV k^−1^ at near room temperature with a decreasing trend observed with the increasing operation temperature (Figure 1b). Electrical conductivity increases slightly with the operation temperature from 55 kS m^−1^ at 26 °C to nearly 60 kS m^−1^ at 44 °C (Figure 1b). The reproducibility could be referred to Table S1 (Supporting Information). The optimal power factor of 2500 µW m^−1^ K^−2^ is seen at near room temperature (Figure 1c). In addition, the thermal conductivity of Ag_2_Se and Nylon film as a whole was measured using the laser flash analysis (LFA) with an average density of 1.6 g cm^−3^ and it is nearly stable in this temperature range with a value of ≈0.56 W m^−1^ K^−1^ (Figure S4a, Supporting Information). However, this measurement reflects the average thermal behavior of the bilayer structure by treating it as a homogeneous material, and thus does not represent the intrinsic thermal conductivity of Ag_2_Se. Further discussion is provided in the supporting information.

We attribute the improved TE performance, particularly the enhanced Seebeck coefficient, to an increased energy filtering effect at GBs. The effectiveness of EF at GBs is governed by both the GB density and the height of the associated energy barriers. As shown in Figure 1d, the EF is weak when the fusing temperature is below the phase transition point, where Ag_2_Se is not in its superionic phase. In this regime, the material lacks fluid‐like deformability, preventing the formation of a sufficient number of GBs, thereby reducing the EF. Conversely, excessively high fusing temperatures also weaken the EF due to grain growth, which reduces GB density. Additionally, the improved crystal connectivity lowers the energy barriers at GBs, further diminishing the EF.

This mechanism is first proven by morphology analysis. Below the phase transition temperature, where Ag_2_Se remains non‐superionic, nanoparticles did not fuse effectively (Figure 1e), resulting in poor interparticle contacts. While fusing above the phase transition temperature enabled effective grain fusion, yielding a dense surface morphology. In addition, enhancing fusing temperature to 200 °C significantly improves the grain merging as seen in Figure 1e.

X‐ray diffraction (XRD) analysis reveals structural reorientation in films fused at 200 °C (Figure 1f), which is not observed in those fused at 150 °C. Grain size calculations based on the Scherrer equation (Figure 1g) indicate grain growth with increasing fusing temperature. These results indicate that increasing the fusing temperature to 200 °C enhances the fluidity of Ag_2_Se, thereby promoting grain merging and structural reorientation.

In addition to structural characterization, the transport properties were systematically measured and compared (Figure S5a, Supporting Information), electrical conductivity increases with fusing temperature, which is attributed to improved grain fusion at higher temperatures, resulting in reduced porosity and enhanced grain connectivity. The film fused at 100 °C shows the lowest Seebeck coefficient, consistent with values reported in the literature.^[^ ^49^ ^]^ In contrast, films fused at 150 °C exhibit the highest Seebeck coefficient (‐215 µV K^−1^), while further increasing the fusing temperature leads to a decline (Figure S5b, Supporting Information).

Carrier concentration and mobility were measured using the Hall effect. Carrier concentration increases with fusing temperature and then stabilizes (Figure 1h). We speculate that the observed increase in carrier concentration is mainly attributed to the reduced porosity, which affects Hall resistance through changes in sample geometry. Higher porosity lowers the number of conducting electrons per unit volume, thus reducing the measured carrier density. Nonetheless, local variations in stoichiometry,^[^ ^50^ ^]^ along with differences in surface,^[^ ^51^ ^]^ interface,^[^ ^52^ ^]^ and impurity^[^ ^53^ ^]^ conditions introduced during the fusing process, may also influence carrier concentration, and their individual contributions cannot be isolated from this single measurement.

Mobility also increases with fusing temperature (Figure 1h). The lowest mobility is observed in samples fused at 100 °C, due to ineffective fusion and the presence of a porous structure, which severely scatters charge carriers. At elevated temperatures, the activation of the superionic state enables effective fusion, reducing porosity and enhancing grain interconnection. This reduces carrier scattering and improves mobility. The different temperature dependances of mobility and Seebeck coefficient suggest that higher temperatures promote grain merging but reduce energy barriers at GBs, due to enhanced interconnectivity. The reduced mobility in films fused at 100 °C is primarily due to high energy barriers at grain‐air interfaces, which scatter electrons across the entire energy spectrum (Figure S6, Supporting Information). In this case, no EF is present because the required asymmetry in the transport distribution function *σ*(*E*), as described in Equation (2), is lost.

Compared to the TE performance of previously reported Ag_2_Se films, our low‐temperature fused Ag_2_Se film shows a record high value of Seebeck coefficient of ‐215 µV K^−1^ at room temperature (compared to ‐140 µV K^−1^ for Ag_2_Se film from hot pressed Ag_2_Se nanowire^[^ ^49^ ^]^ and ‐180 µV K^−1^ for Ag_2_Se film prepared from sputtering),^[^ ^54^, ^55^, ^56^ ^]^ and achieve a high power factor at the same time (Figure 1i). Although several previous studies reported a slightly higher power factor of Ag_2_Se with the introduction of excess crystalline Ag phase or other dopants, significant reduction in Seebeck coefficient was associated,^[^ ^55^, ^56^, ^57^ ^]^ implying that the enhancement of power factor in these studies was a consequence of enhanced electrical conductivity only (Figure S7, Supporting Information). In contrast, the power factor in our samples is enhanced mainly from the significantly increased Seebeck coefficient through EF with minor effect on electrical conductivity, which is more advantageous because the electrical conductivity is also associated with thermal conductivity.

### 2.1 Low Temperature Fusing Process

**Figure 2:**
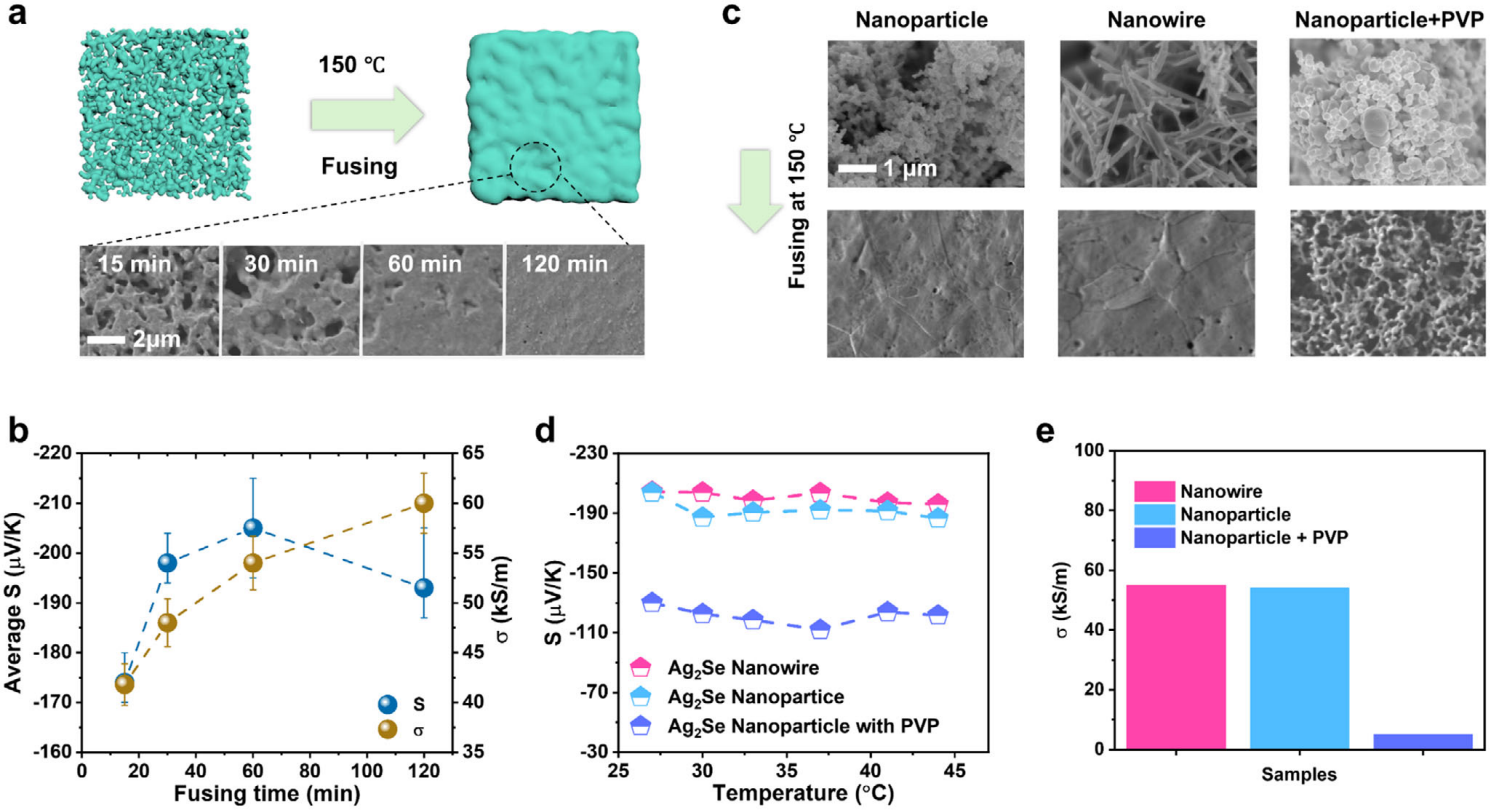
Low temperature fusing process. a) The evoluation of surface morphology with respect to the fusing time (under the same temperature 150 °C). b) Electrical conductivity and Seebeck coefficient with respect to fusing time. c) The SEM images of 3 types of Ag_2_Se films (made from Ag_2_Se nanoparticles, Ag_2_Se nanowires, and nanoparticles with PVP respectively) before and after the fusing process. d) Seebeck coefficient with repect to operation tempertures of these 3 types of Ag_2_Se films. e) Electrical conductivity of 3 types of Ag_2_Se films at room temperature.

To further investigate this low temperature fusing process, Ag_2_Se films fused at 150 °C with different fusing time were prepared. **Figure** **2**a illustrates the evolution of the surface morphology of the Ag_2_Se films over fusing time. Effective fusing already started as short as 15 min, evidenced by the merging of numerous small grains. As the fusing time increases, Ag_2_Se particles fill pores and voids first, nearly completes the fusing at ≈ 60 min, and continuously smooths the surface morphology at further extended fusing time. The Seebeck coefficient and electrical conductivity of the fused Ag_2_Se films at various fusing times were measured accordingly (Figure 2b). The electrical conductivity increases monotonically with the fusing time, primarily due to the reduction in porosity and better merging process. While the average Seebeck coefficient (from 25 to 45 °C) increases with the fusing time but achieves its highest value at 60 min and then slightly decreases at further extended fusing time. This behavior is consistent with the morphology observation that nearly all Ag_2_Se nanoparticles are fused at 60 min.

From the perspective of the EF effect, a short fusing time results in a porous film, which cannot produce a strong EF due to the lack of intensive GBs. As the fusing time increases, the film becomes denser, and the number of pores decreases. This densification leads to the formation of numerous effective GBs. Consequently, enhanced EF contributes to an increased Seebeck coefficient. However, excessive fusing at prolonged times improves grain connectivity, reducing the energy barriers at GBs. As a result, the Seebeck coefficient decreases due to the weakened EF. This reduction in EF is accompanied by increased electrical conductivity, driven by enhanced carrier mobility.

The XRD patterns of all samples fused at different durations are compared (Figure S8a, Supporting Information). No significant differences are observed. The grain sizes of Ag_2_Se are analyzed across different fusing times (Figure S8b, Supporting Information). It remains nearly constant over the entire time range except a slight growth at 120 min, supporting the conclusion that the low temperature fusing process is driven by fluid‐like plastic deformation, and it enhances the connections or bonding between grains without causing significant crystal structural change or growth of grains.

In addition to the fusing temperature and time, the effects of surfactants and the morphology of raw Ag_2_Se materials were investigated. Three types of samples, namely Ag_2_Se film made of Ag_2_Se nanoparticles, Ag_2_Se film made of Ag_2_Se nanowires, and Ag_2_Se film made of Ag_2_Se nanoparticles covered with Polyvinylpyrrolidone (PVP), which served as a surfactant to stabilize nanoparticles and enhance their dispersion in the solvent, were prepared for comparison. It is observed that both Ag_2_Se nanoparticles and nanowires have been effectively fused (Figure 2c). The “bigger grains” observed in Ag_2_Se nanowires after fusing, which is distinct with nanowires, is attributed to fluid‐like plastic deformation, which leads to the merging of nanowires. Recrystallization does not occur during this process, as the temperature remains well below the melting point. This is further supported by the consistent XRD patterns of Ag_2_Se nanowires before and after fusing (Figure S9, Supporting Information). However, the Ag_2_Se with PVP surfactants could not fuse effectively under the same conditions, resulting in a porous morphology (Figure 2c).

The Seebeck coefficients of three samples are compared (Figure 2d). Both the fused Ag_2_Se nanoparticles and nanowires exhibit a high Seebeck coefficient of ≈‐210 µV K^−1^, but the Ag_2_Se with PVP yields a much lower value of ≈‐130 µV K^−1^ at room temperature. Again, this proves the strong impact of the successful fusing process on improving Seebeck coefficient (due to enhanced density of GBs). In addition, high electrical conductivity is observed for both fused Ag_2_Se nanoparticles (55 kS m^−1^) and fused Ag_2_Se nanowires (54 kS m^−1^) while the addition of PVP results in a significantly low electrical conductivity (5 kS m^−1^) (Figure 2e).

These findings suggest that the fusing process plays a critical role in improving Seebeck coefficient and electrical conductivity. A low temperature fusing will not only create many GBs but also preserve the high energy barrier on GBs to enhance the Seebeck coefficient via energy filtering effect and increase the electrical conductivity via close contacts between nanoparticles at the same time. This process is not affected by the morphology of raw materials but suppressed by the addition of surfactants (the similar phenomenon is observed in references^[^ ^58^, ^59^ ^]^), suggesting the fusing process is realized by fluid‐like plastic deformation induced by mobile Ag ions rather than simple densification via reducing pores.

### 2.2 Composition and Grain Boundaries

**Figure 3:**
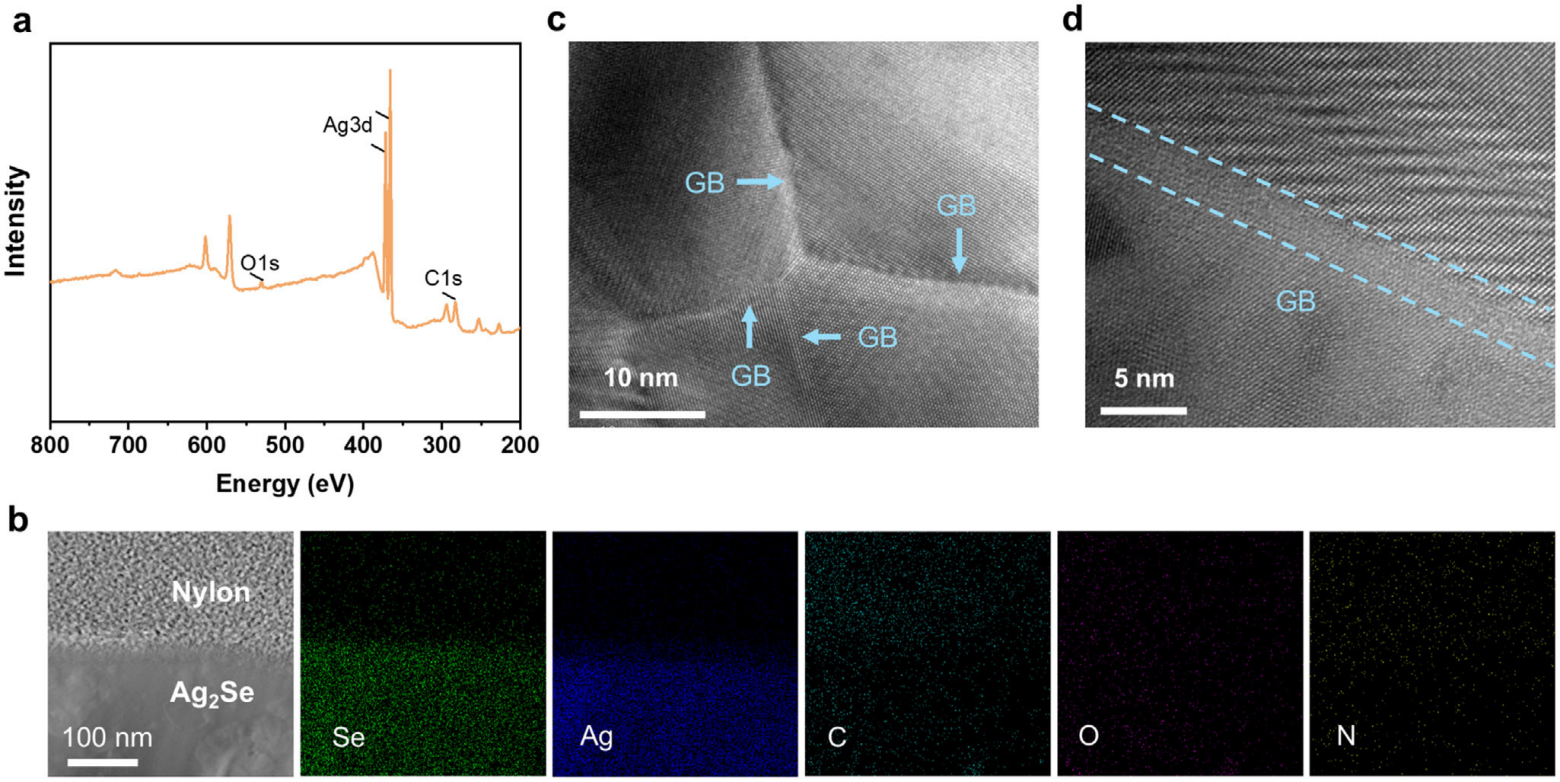
Composition and grain boundaries. a) XPS survey spectrum of Ag_2_Se nanoparticles. b) EDS elemental mapping of the fused Ag_2_Se film near the boundary between Ag_2_Se and Nylon in TEM. c) The TEM image of the morphology of GBs with intergranular lattice matching. d) GBs in fused Ag_2_Se with disordered area.

Composition analysis of Ag_2_Se nanoparticles is essential, as it reveals the chemical nature of GBs. Elemental compositions were quantitatively determined using energy‐dispersive X‐ray spectroscopy (EDS) in SEM (Table S2, Supporting Information), and the corresponding element mapping is shown in Figure S10a (Supporting Information). However, the detection of a high carbon content without accompanying oxygen is questionable, likely resulting from chamber contamination rather than actual sample composition. To further investigate the carbon content, X‐ray photoelectron spectroscopy (XPS) was performed. The XPS survey spectrum shows minor signals from oxygen and carbon, with a dominant signal from Ag (**Figure** **3**a). Considering potential surface contamination, it can be reasonably concluded that the synthesized Ag_2_Se nanoparticles are clean and free from significant adsorption of extraneous substances.

To investigate the composition of the fused Ag_2_Se films, EDS elemental analysis (Table S2, Supporting Information) and elemental mapping (Figure S10b, Supporting Information) were performed using SEM. The results show that the fusing process does not alter the composition, and Ag and Se elements remain uniformly distributed, excluding the influence of carbon contamination. Furthermore, Table S2 (Supporting Information) shows that films fused at various temperatures (up to 200 °C) maintain consistent composition, indicating that elevated fusing temperatures do not affect the elemental makeup. These findings confirm that the entire fusing process does not alter the composition of the Ag_2_Se films.

To obtain in‐depth observations of GBs, a focused ion beam (FIB) was used to prepare a lamella from the fused Ag_2_Se film for transmission electron microscopy (TEM) analysis. Elemental mapping reveals a distinct interface between the Ag_2_Se film and the underlying Nylon substrate, along with a uniform distribution of Ag and Se in the fused Ag₂Se film (Figure 3b). Additionally, composition analysis (Table S3, Supporting Information) aligns with previous results, indicating only trace amounts of carbon and oxygen.

TEM images reveal numerous GBs of different configurations, which serve as effective energy filters (Figure 3c,d). Some GBs exhibit sharp transitions with well‐aligned crystal structures, indicating strong intergranular lattice matching (Figure 3c). In contrast, other GBs display disordered regions (Figure 3d), which can be attributed to the low‐temperature fusing process that limits crystallization near the boundaries. These disordered GBs present high energy barriers, contributing to the observed energy filtering effect.

### 2.3 Role of Excess Ag in Fusing Process

To study the effect of excess Ag on the fusing process and TE performance, Ag_2_Se nanoparticles were synthesized with various reaction times in AgNO_3_. It is expected that the resultant Ag_2_Se films after fusing process would have different Ag contents correspondingly, with a longer reaction time yielding a larger Ag content. Three types of Ag_2_Se films, with synthesis reaction times of 2, 24, and 48 h, were prepared following the method detailed in the Methods section and are labeled as R2, R24, and R48, respectively. It is worth noting that our discussion in the previous section is based on R48, corresponding to a 48‐h reaction duration, which is the best performing sample.

**Figure 4:**
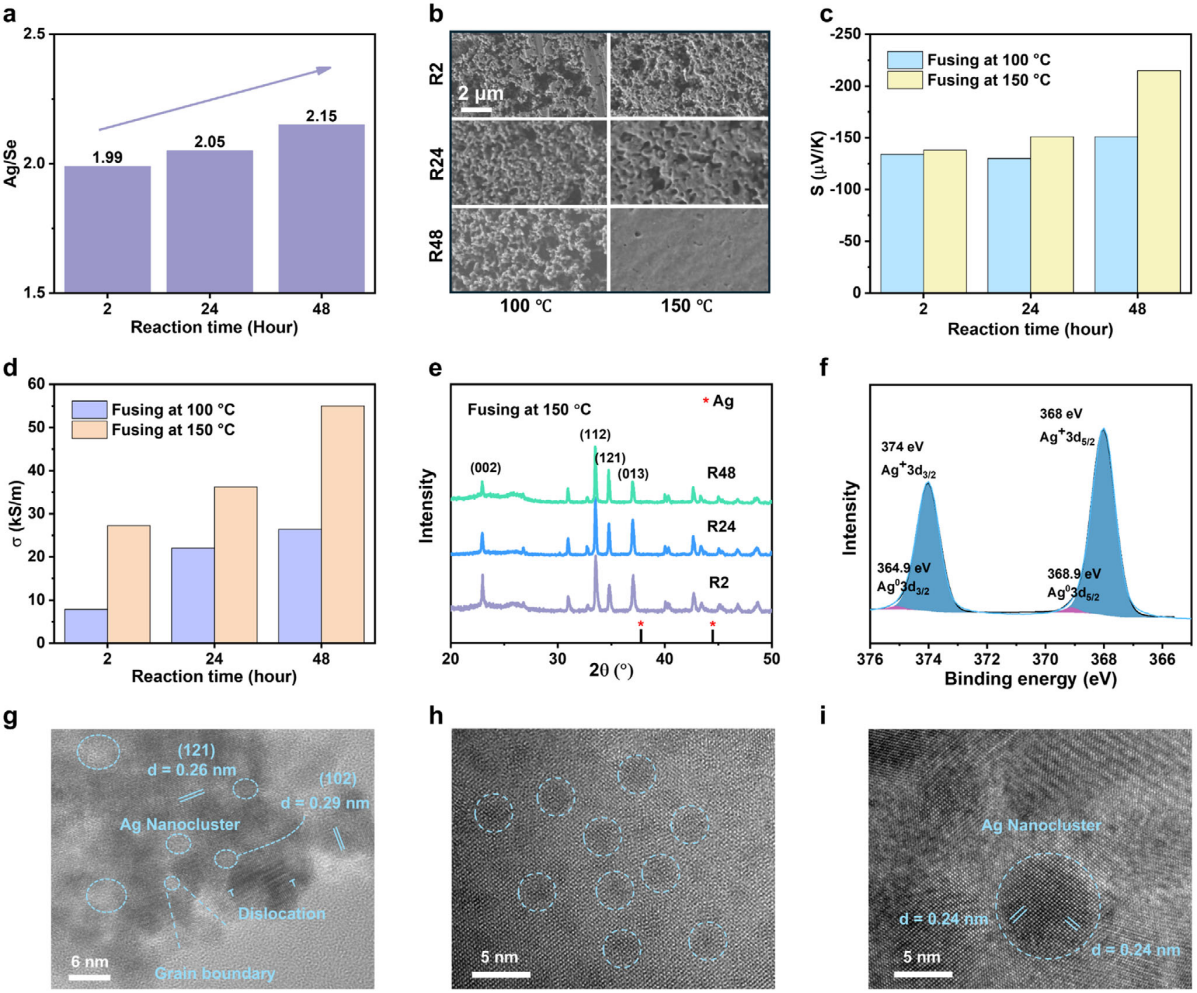
Effect of excess Ag on fusing process. a) Ag/Se ratio with respect to the reaction time. b) Comparison of the surface morphology of Ag_2_Se films from nanoparticle samples of different reaction times. c) Seebeck coefficient and d) electrical conductivity of the Ag_2_Se film fused at 100 and 150 °C with respect to the reaction time during the synthesis of Ag_2_Se nanoparticles. e) XRD pattern of the Ag_2_Se films fused from R2, R24, and R48. f) XPS spectra of Ag element in R48 sample. g) Transmission electron microscopy (TEM) image of Ag_2_Se nanoparticles of as‐synthesized R48. TEM images of fused R48 film (150 °C) with uniformly distributed (h) amorphous small Ag nanoparticles and (i) crystallized Ag nanoparticles.

EDS elemental analysis reveals that the Ag content increases with longer reaction times (**Figure** **4**a), and the detailed compositions are listed in Table S4 (Supporting Information). Furthermore, comparison of the XRD patterns of annealed Ag_2_Se powders shows that Se nanoparticles are fully converted into Ag_2_Se after 2 h of reaction (Figure S11, Supporting Information), consistent with previous reports in the literature.^[^ ^46^ ^]^ Therefore, varying the reaction time primarily affects the Ag content.

Figure 4b compares the morphology of these films under two different fusing conditions. It is not surprised that there is no observable fusing effect for all samples fused in the non‐superionic state (100 °C). However, even under above‐mentioned optimal fusing temperature (150 °C), at which samples are supposedly in the superionic state, only R48 sample exhibits effective fusing and shows a smooth surface, whereas the fusing effect is minimal in R2 and R24 samples, indicating a critical role of particle synthesis time (or Ag content) on the effectiveness of fusing process.

Their TE performance was also measured for comparison. Electrical conductivity is found to increase with reaction times in general, but for the samples with the same reaction time, those fused in the superionic state exhibit significantly higher electrical conductivity than those fused in the non‐superionic state (Figure 4c). All samples with insufficient fusing (regardless of the fusing temperature or reaction time) exhibit a similar Seebeck coefficient of ≈‐140 µV K^−1^ (Figure 4d). However, a significant increase in the Seebeck coefficient is observed for R48 when fused at 150 °C, while this is not observed in the case of R2 and R24. This builds a strong correlation between the TE enhancement and fusing effectiveness: poor grain contacts or large porosity in insufficient fused samples lead to fewer GBs and a weak energy filtering effect, limiting the improvement in the Seebeck coefficient (Figure S6, Supporting Information).

Therefore, we conclude that higher Ag content facilitates the fusing process, which in turn simultaneously enhances both electrical conductivity and the Seebeck coefficient. This enhancement in electrical conductivity arises from reduced porosity and contact resistance caused by improved fusing, while the increase in Seebeck coefficient is attributed to a high density of effective GBs, which strengthens the energy filtering effect. Rather than donating electrons directly to the Ag_2_Se matrix, given the extremely low solubility of Ag.^[^ ^24^ ^]^

The mechanism by which Ag nanoclusters facilitate the fusing process can be summarized as follows. First, amorphous Ag nanoclusters are highly deformable,^[^ ^60^ ^]^ allowing them to accommodate strain without inducing interfacial cracking between the Ag and Ag_2_Se matrix. Second, they induce local lattice distortions, which can serve as nucleation sites for dislocations or twin boundaries, both of which are known to enhance plastic deformation.^[^ ^61^, ^62^ ^]^ Third, Ag nanoclusters may act as reservoirs of Ag atoms, shifting the equilibrium toward a higher Ag⁺ concentration. Subsequent diffusion of Ag⁺ into interstitial sites may weaken Ag–Se bonding and slightly reduce overall lattice rigidity, thereby promoting plastic flow.^[^ ^24^ ^]^

Further analyses were conducted to examine the state of Ag in Ag_2_Se. No significant differences are observed in the XRD patterns of R2, R24, and R48 (Figure 4e). More importantly, no crystalline Ag phase is detected in all XRD patterns, indicating that Ag exists not as large crystalline phases but as small nanoclusters. XPS analysis (Figure 4f) confirms the presence of a small amount of Ag^0^, indicating that Ag does not undergo self‐doping into the Ag_2_Se crystal matrix. This conclusion is further proven by transmission electron microscopy (TEM) image of Ag_2_Se nanoparticles, revealing some nanoclusters (Figure 4g) that can be attributed to Ag. Thus, the excess Ag is present as uniformly distributed nanoclusters embedded in the Ag_2_Se matrix, which do not show crystal signal in the XRD patterns.

For the films after fusing, two types of Ag nanoparticles are identified: smaller amorphous particles (Figure 4h) and larger crystallized particles (Figure 4i). The smaller amorphous Ag nanoparticles are consistent with those observed before fusing, while the formation of larger crystallized nanoparticles is attributed to the merging of smaller particles induced by the fusing process. However, most of Ag is present in the form of amorphous nanoclusters with a few crystallized Ag observed.

We further increased the Ag content by using a strong reducing agent during Ag_2_Se nanoparticle synthesis, which resulted in the presence of crystalline Ag phase in Ag_2_Se matrix, as shown in XRD pattern (Figure S12, Supporting Information). It is found that effective fusing could not be achieved in such a sample even under above‐mentioned optimal fusing conditions, evidenced by its porous morphology (Figure S13, Supporting Information). It seems that ultrafine and uniform‐distributed nanoclusters embedded in Ag_2_Se matrix is a requirement for promoting effective fusing of Ag_2_Se nanoparticles in low‐temperature superionic state.

### 2.4 Mechanism Discussion

**Figure 5:**
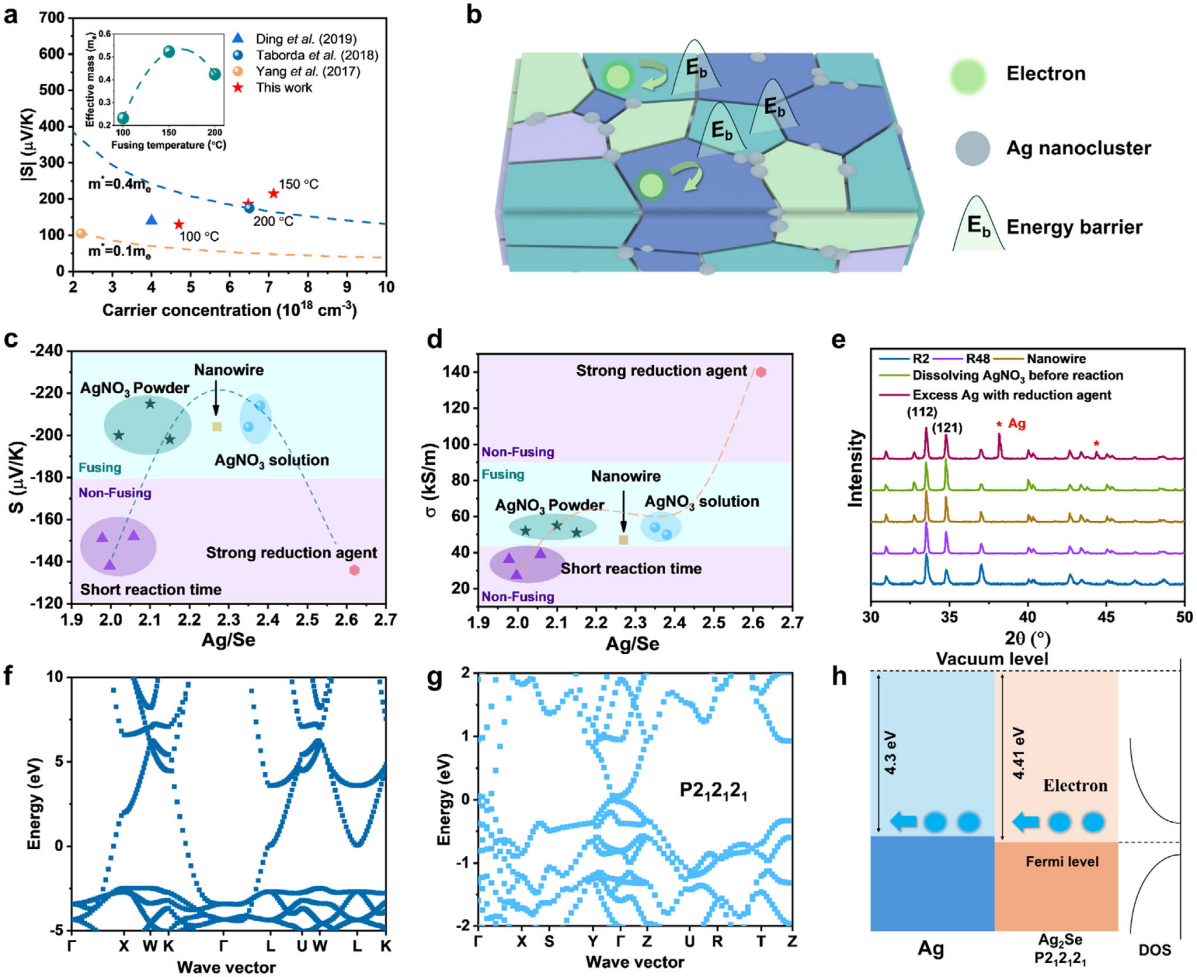
Mechanism analysis. a) Pisarenko plot (Seebeck coefficient with respect to carrier concentration). b) Schematically showing the energy filtering effect from grain boundaries. c) Seebeck coefficient and d) electrical conductivity with respect to the Ag/Se ratio of Ag_2_Se films synthesized under different experimental conditions. e) The XRD patterns of Ag_2_Se films synthesized under different experimental conditions. Band structure of f) Ag and g) P2_1_2_1_2_1_ Ag_2_Se. h) Schematically showing the energy alignment between Ag and P2_1_2_1_2_1_ phase of Ag_2_Se.

The Pisarenko plot (**Figure** **5**a) is constructed using reported data as references, in which Ag_2_Se is shown to have an effective mass of 0.1^[^ ^63^ ^]^ and 0.4 *m_e_*,^[^ ^54^ ^]^ respectively. It is observed that the Ag_2_Se film fused at 100 °C exhibits an effective mass similar to that of previously reported hot‐pressed Ag_2_Se films,^[^ ^49^ ^]^ falling between 0.1 and 0.4 *m_e_*. In contrast, the Ag_2_Se film fused at 150 °C shows an abnormally high effective mass exceeding 0.4 *m_e_*. Since no intentional band structure modification was introduced to increase the effective mass, this suggests that the simplified single parabolic band (SPB) model with energy‐independent scattering (Equation (3)) is inadequate. Instead, the full expression for the Seebeck coefficient considering energy‐dependent scattering (Equation (4)) should be applied. These results indicate that the Ag_2_Se films fused at 150 °C exhibit a strong energy filtering effect, leading to energy‐dependent scattering, which is not observed in the films fused at 100 °C. In addition, fusing at higher temperatures (200 °C) yields a reduced effective mass (inset of Figure 5a), an indication of the fusing dependent barrier potential, suggesting that over fusing may reduce energy dependent scattering.

(3) $$S=\frac{8\pi^{2}}{3}\frac{{k_{B}}^{2}T}{q\hbar^{2}}m^{*}{\left(\frac{\pi }{3n}\right)}^{\frac{2}{3}}$$

(4) $$S=\frac{\pi^{2}}{3}\frac{{k_{B}}^{2}}{q}T\left(\frac{1}{n}\frac{\partial n}{E}+\frac{1}{\mu }\frac{\partial \mu }{E}\right){|}_{E=E_{f}}$$

Where *k_B_* is Boltzmann constant, *q* represents charge, ℏ is Planck constant, *n* is carrier density *µ* is mobility, *E* refers to energy and *E_f_* is Fermi energy.

Since XRD analysis confirms that the crystal structure of Ag_2_Se remains the same in various samples, the improved Seebeck coefficient may originate from energy filtering induced by GBs or Ag nanoclusters rather than crystallinity improvement or structural variation. Here, we propose that the primary mechanism behind the improved Seebeck coefficient is caused by energy barriers at dense GBs (Figure 5b), rather than Ag nanoclusters. As discussed earlier, sufficient fusing will bring grains in close contact, and thus increases the density of GBs, but further fusing will cause the grain growth and inter‐grain diffusion that reduce the density of GBs and the strength of energy barrier at GBs. Both sets of experiments on fusing temperature (Figure S5b, Supporting Information) and fusing time (Figure 2b) follow exactly such a trend on the Seebeck coefficient, supporting the GB assumption.

To understand the role of Ag nanoclusters beyond facilitating the fusing process, other types of Ag_2_Se nanoparticles or nanowires with different experimental conditions were synthesized. For each experiment, one of these modified conditions was implemented while the remaining parameters were maintained as described in the Methods section. The synthesis conditions for each sample are detailed in Table S5 (Supporting Information). Ag_2_Se films were prepared through fusing at 150 °C under the pressure of 1 MPa. Figure 5c presents the Seebeck coefficient of these films as a function of the Ag/Se ratio (determined via elemental analysis) at room temperature. Ag_2_Se films with shorter reaction times (less than 24 h) exhibit a low Ag/Se ratio and Seebeck coefficient, as effective fusing is not observed in these samples. Extending the reaction time to 48 h increases the Ag/Se ratio, and Ag‐facilitated effective fusing results in nearly a 50% enhancement in the Seebeck coefficient. Additionally, dissolving AgNO_3_ before the reaction significantly increases the Ag/Se ratio, leading to the formation of more Ag nanoclusters. This promotes effective fusing and enhances the Seebeck coefficient. However, across a wide Ag/Se ratio range (≈2.1 to 2.4) from the samples that have been effectively fused, the Seebeck coefficient remains nearly constant. This indicates that TE enhancement or energy filtering does not originate from Ag nanoclusters. A similar conclusion could be obtained from the electrical conductivity (Figure 5d). Samples synthesized with shorter reaction times exhibit the lowest electrical conductivity, as the lack of a fusing effect and the presence of pores hinder electron transport. The fusing effect promotes a more compact surface structure, leading to improved electrical conductivity. Consequently, Ag_2_Se films with an Ag/Se ratio ranging from 2.1 to 2.4, where effective fusing is seen, exhibit a similar electrical conductivity of ≈55 kS m^−1^. This plateau in electrical conductivity further suggests that Ag nanoclusters have a minimal energy filtering effect, as an increase in Ag content does not significantly enhance electron scattering.

The XRD patterns (Figure 5e) reveal that only the samples prepared with a strong reducing agent exhibit distinct Ag peaks, whereas merely extending the reaction time does not result in the formation of largely agglomerated Ag phases. This indicates that the strong reducing agent facilitates the formation of interconnected crystalline Ag phases. Although it could increase the Ag/Se ratio significantly to 2.6, the formation of a percolative Ag secondary phase reduces the Seebeck coefficient (Figure 5c) due to a substantial increase in electrical conductivity (Figure 5d), suggesting crystalline Ag is not the cause of energy filtering even they exist as second phases, which is different from previous studies in other TE materials.

To gain deeper insight into the underlying mechanisms, computational analysis was performed. β‐Ag₂Se is known to adopt orthorhombic structures, corresponding to the space groups P2_1_2_1_2_1_ at ambient condition, while Ag is a metal material. The gapless band structure of Ag indicates its metallic behavior (Figure 5f) with electrons near Fermi level participating transportation process. In comparison, β‐Ag₂Se is a semimetal without considering spin orbital coupling (SOC) (Figure 5g) while SOC will lead to a small bandgap.^[^ ^29^ ^]^ In addition, the work function of Ag and β‐Ag₂Se are calculated and compared (Figure 5h). It is a metallic contact with matchable work function. Therefore, the EF from Ag nanoclusters is ruled out theoretically.

### 2.5 Applications

**Figure 6:**
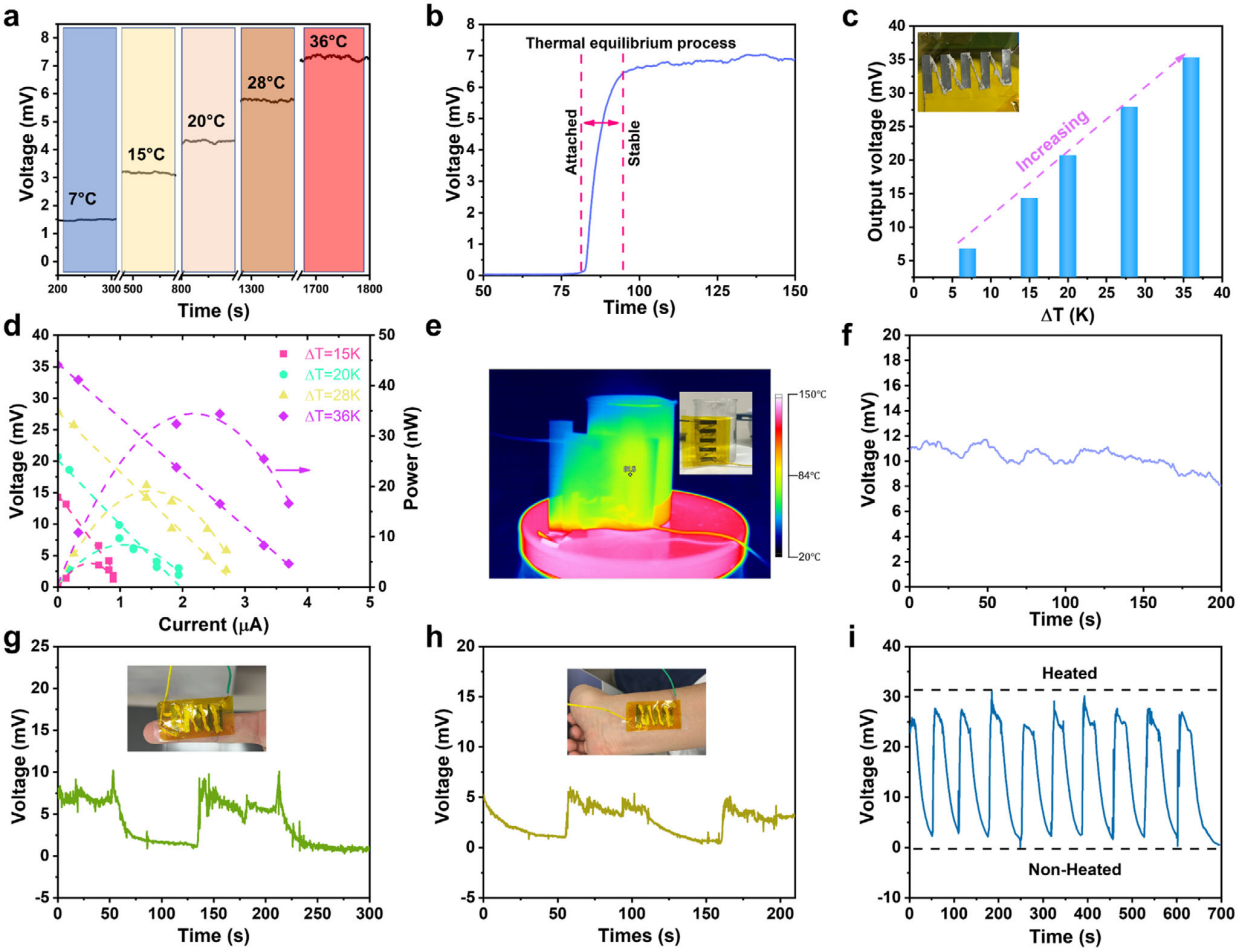
Output power analysis and applications. a) Voltage output with temperature differences for a single TE device. b) The response time of a single Ag_2_Se device. c) Output voltage with respect to temperature difference of Ag_2_Se TE module. d) The output power measurement of Ag_2_Se TE module at different temperature differences. e) Demonstration of the heat harvesting ability from hot water and f) corresponding output voltage with respect to time. The TE energy harvesting demonstration from (g) finger and (h) wrist. i) Cyclic heating and cooling of the Ag_2_Se TE module.

To evaluate the thermoelectric performance of the fabricated Ag_2_Se film for practical applications, a series of tests was conducted. The output voltage of a single Ag_2_Se device remained stable at a given temperature gradient and linearly increased with the temperature gradient (**Figure** **6**a). It showed a rapid response to external heat stimulation, with ≈12 s for the voltage to stabilize when placed on a hot plate at 60 °C (Figure 6b).

Five such Ag_2_Se stripes were then serially connected to construct an energy‐harvesting module. The output voltage of the module increased nearly linearly with the temperature gradient, reaching a maximum value of ≈35 mV at a temperature difference of 36 K (Figure 6c). The output power of the module was also tested and calculated at various loading resistors. A maximum output power of 35 nW was observed at a temperature difference of 36 K (Figure 6d). This module was then attached to a cup containing hot water. Infrared imaging confirms that the entire TE module was uniformly heated (Figure 6e), and a stable voltage output of 10 mV was observed (Figure 6f), demonstrating its capability in harvesting low‐temperature waste heat. This TE module was also tested on a finger (Figure 6g) and wrist (Figure 6h), ≈7 mV and 5 mV voltage outputs were produced, respectively, indicating the possibility to harvest body heat for powering wearable electronics. Finally, the cyclic heating and cooling tests were conducted to check its reliability, and the module exhibited repeatable and stable output (Figure 6i).

## 3 Conclusion

A superionic‐induced fluid‐like plastic deformation mechanism has been successfully utilized to fuse Ag_2_Se nanoparticles at a low temperature. It is found that this low temperature fusing process not only effectively fuse Ag_2_Se nanoparticles to a dense film but also helps preserve high density of effective GBs, enhancing energy filtering effect. In addition, excess Ag nanoparticles work as fusing agents facilitating this fusing process. As a result, this strong energy filtering effect significantly enhances the Seebeck coefficient, reaching a record‐high value of ‐215 µV K^−1^. This improvement, combined with the enhanced electrical conductivity, contributes to a high power factor of 2500 µW m^−1^ K^−2^ at room temperature. Consequently, these enhancements lead to a remarkable thermoelectric performance, indicating its practical potential in harvesting near room‐temperature waste heat.

## 4 Experimental Section

### Materials

Silver nitrate (AgNO_3_, >99%, Sigma‐Aldrich), Selenium oxide (SeO_2_, 98%, Sigma‐Aldrich), L‐Ascorbic acid (>99%, Sigma‐Aldrich), β‐Cyclodextrin (>97%, Sigma‐Aldrich), and Ethanol glycol (EG, Sigma‐Aldrich).

### Synthesis of Selenium (Se) Nanoparticles

SeO_2_ (0.25 g) and β‐Cyclodextrin (0.25 g) were dissolved in deionized (DI) water (50 mL), and the solution was stirred at 350 rpm for 10 min to form solution A. L‐Ascorbic acid was dissolved in DI water (50 mL) and stirred at 350 rpm for 10 min to form solution B. Solution A was then added to solution B while stirring at 350 rpm. The color of the mixed solution changed to red instantly, followed by the formation of small red aggregates. The mixture was left to react for 4 h. The resulting Se nanoparticles were washed three times with DI water followed by two washes with ethanol and then stored in ethanol solution for use.

### Synthesis of Se Nanowires

The washed Se nanoparticles were dissolved in ethanol (50 mL) and sonicated for 1 min to form the seeds of hexagonal Se. The solution was then aged 24 h to allow for the growth of Se nanowires.

### Synthesis of Ag2Se Nanoparticles

The as‐prepared Se nanoparticles were dissolved in ethylene glycol (EG) (50 mL) and sonicated for 5 min. Subsequently, AgNO_3_ powders (1.5 g) were slowly added to the Se dispersion while stirring at 350 rpm. The reaction was allowed to proceed for 48 h to yield Ag_2_Se nanoparticles. The Ag_2_Se nanoparticles were then washed twice with ethanol and twice with DI water. Finally, the Ag_2_Se nanoparticles were washed once more with ethanol and dispersed in ethanol (40 mL).

### Synthesis of Ag2Se Nanowires

The Se nanowires were dispersed in EG (50 mL) and sonicated for 5 min. Subsequently, AgNO_3_ powders (1.5 g) were added to the Se dispersion while stirring at 350 rpm for reaction, followed by washing with ethanol and DI water. Finally, the Ag_2_Se nanowires were dispersed in ethanol (40 mL).

### Preparation of the Fused Ag2Se Film

A dispersion of either Ag_2_Se nanoparticles or Ag_2_Se nanowires (10 mL) was vacuum filtered onto a nylon membrane to form a porous film with loosely bonded particles. The film was then dried under ambient conditions for 24 h and was subsequently pressurized at 1 MPa and heated to 150 °C (slightly above the phase transition temperature where it exhibits superionic behavior) for 1 h. Finally, the film was cut into various shapes for characterization and device fabrication.

### Thermoelectric Parameters Measurements

The Seebeck coefficient was measured using a custom‐made setup. One side of the film was placed on a hot plate with a controllable temperature, while the other side was suspended in the air. The film was connected to a Metrohm Autolab (for recording open‐circuit voltage) using wires and silver paste, while the temperature was measured with a two‐channel thermocouple. Electrical conductivity was measured using an Ossila four‐point probe. For electrical conductivity measurements as a function of temperature, a heater was placed beneath the film, utilizing the Ohmic heating effect. The temperature was controlled by adjusting the voltage, and the temperature was recorded using a thermocouple. Thermal conductivity was measured using an LFA 467 HT Hyperflash apparatus.

### Materials Characterization

X‐ray diffraction (XRD) was performed using a Bruker D2 Phaser with a Cu K‐α line (1.54184 Å). Focused ion beam (FIB) lamella preparation was conducted on Carl Zeiss Crossbeam 350. The morphology of Ag_2_Se was examined using a scanning electron microscope (SEM, JEOL JSM‐7610F) and a transmission electron microscope (TEM, Tecnai TEM 200 kV). Atomic force microscopy (AFM) images were acquired using an MFP‐3D Origin to assess the surface roughness of the films. Elemental analysis was performed using JEOL JSM‐7610F to analyze the composition of the elements. X‐ray photoelectron spectroscopy (XPS) measurements were conducted using an Escalab Xi+ system. Carrier concentrations were measured with Ecopia HMS‐5000 Hall Effect Measurement System. Profiler measurement was conducted on Olympus OLS5100 Profilometer.

### Calculations

Density functional theory (DFT) calculations were performed using the Quantum Espresso software.^[^ ^64^ ^]^ The ultrasoft (US) pseudopotential was employed to simplify the potential energy from core electrons, and the generalized gradient approximation (GGA), formulated by Perdew, Burke, and Ernzerhof (PBE), was used to approximate the exchange‐correlation functional. A plane‐wave basis set with a cutoff energy of 50 Ry and an electron density cutoff of 500 Ry was applied in the self‐consistent field (SCF) calculations, using a K‐point mesh of 8×4×4. The work function was calculated using a 1 × 1 × 4 supercell with a vacuum layer of 20 Å along the z‐direction. The work function was determined by subtracting the Fermi energy from the potential energy in the vacuum.

## Conflict of Interest

The authors declare no conflict of interest.

## Supporting information



Supporting Information

## Data Availability

The data that support the findings of this study are available in the supplementary material of this article.
