# Supplementary material for: Ultrahigh Seebeck Coefficient and Power Factor in Low‐Temperature Fused Ag2Se Films via Superionic‐Driven Plastic Deformation
Source: Adv Sci (Weinh). 2025 Sep 16;12(41):e08381. doi: 10.1002/advs.202508381 (PMC12591161; doi:10.1002/advs.202508381)
Supplement: Supplementary file 1 — Supporting Information [file ADVS-12-e08381-s001.docx]

**Supporting Information**

**Ultrahigh Seebeck Coefficient and Power Factor in Low-Temperature Fused Ag_2_Se Films via Superionic-Driven Plastic Deformation**

*Dezhuang Ji, Baosong Li, Xuan Li, Husam Hashem AlTakrori, Balamurugan Thirumalraj, Moh’d Rezeq, Wesley Cantwell, Lianxi Zheng**

Dezhuang Ji, Lianxi Zheng

Department of Mechanical and Nuclear Engineering, Khalifa University of Science and Technology, P.O. Box 127788, Abu Dhabi, United Arab Emirates

Baosong Li, Wesley Cantwell

Department of Aerospace Engineering, Khalifa University of Science and Technology, P.O. Box 127788, Abu Dhabi, United Arab Emirates

Baosong Li, Balamurugan Thirumalraj, Lianxi Zheng

Research & Innovation Center for Graphene and 2D Materials (RIC-2D), Khalifa University of Science and Technology, P.O. Box 127788, Abu Dhabi, United Arab Emirates

Xuan Li, Lianxi Zheng

Research and Innovation on CO2 and H2 Center (RICH), Khalifa University of Science and Technology, P.O. Box 127788, Abu Dhabi, United Arab Emirates

Husam Hashem AlTakrori, Moh’d Rezeq

Department of Physics, Khalifa University of Science and Technology, P.O. Box 127788, Abu Dhabi, United Arab Emirates

*Corresponding author: lianxi.zheng@ku.ac.ae (Lianxi Zheng)

**Supplementary Text**

**Scherrer equation:**

$$D=\frac{K\lambda}{\beta cos\theta}$$

Where D is grain size, K is Scherrer constant and taken as 0.9, λ is the wavelength of X-ray, β is full width at half maximum (FWHM) and θ is peak position.

**Thermal conductivity:**

Regarding the accuracy of the laser flash (LFA) measurement, it essentially reflects the average thermal behavior of the two-layer structure, treating it as a homogeneous material. In theoretical analysis, Ag_2_Se has its own thermal conductivity (k_1_), thermal diffusivity (η_1_), heat capacity (c_p1_), density (ρ_1_), and thickness (d_1_), which differ from those of Nylon (k_2_, η_1_, c_p2_, ρ_2_, and d_2_). The measurement scheme is illustrated in Figure S4b, where Nylon and Ag_2_Se are thermally connected in series.

For homogeneous materials, the thermal conductivity is typically calculated using the relation: k=c_p_∙η∙ρ with η and c_p_ measured experimentally. According to the transient solution of the heat diffusion model without considering interfacial thermal resistance, the measured thermal diffusivity (η) can be approximately expressed in terms of η_1_ and η_2_, based on the configuration shown in Figure S4b. Specifically, d^2^/η is proportional to d_1_^2^/η_1_ + d_1_^2^/η_2_, using a first-order expansion of the exponential function. In contrast, the expressions for heat capacity (c_p_) and density (ρ) follow a thickness-weighted average: c_p_ = c_p1_∙d_1_+c_p2_∙d_2_ and ρ = ρ_1_∙d_1_+ ρ_2_∙d_2_. Therefore, the measured thermal conductivity is essentially a combined contribution from the two materials.

Since it is difficult to obtain a free-standing Ag_2_Se film, the Ag_2_Se on Nylon composite is used as a whole in practical applications. As a result, the thermal properties of the composite influence the temperature difference between the two ends of the film, which in turn affects the thermoelectric energy conversion efficiency.


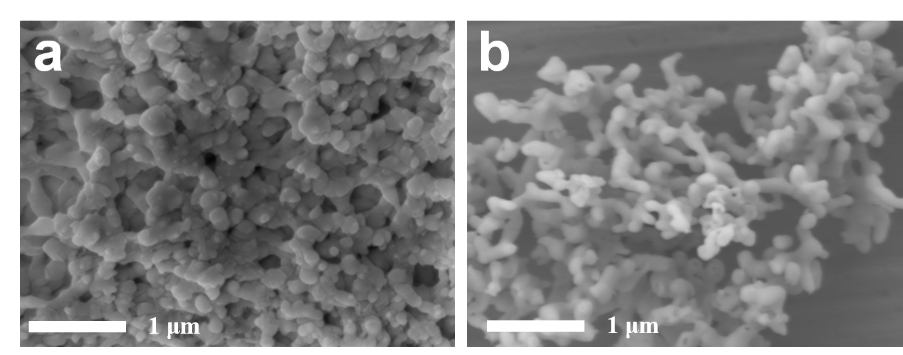


Figure S1: Scanning electron microscopy (SEM) images of (a) Se nanoparticles and (b) Ag_2_Se nanoparticles.


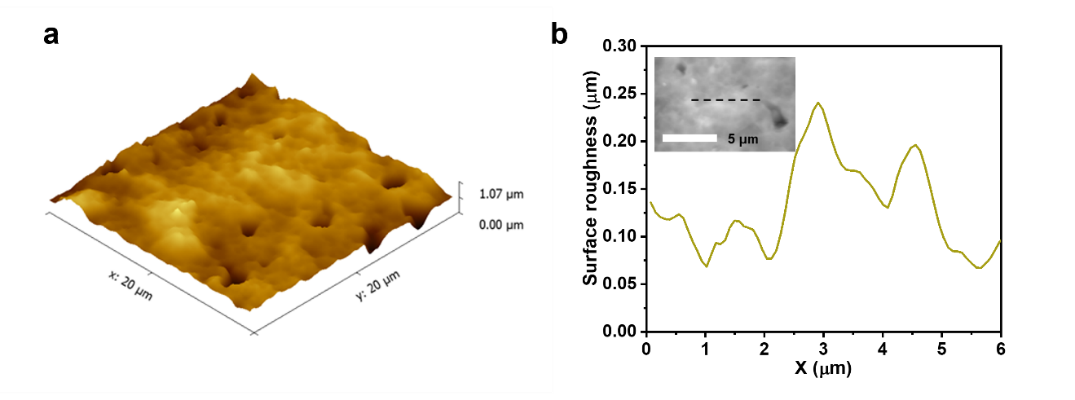


Figure S2: (a) The atomic force microscopy (AFM) image and (b) surface roughness of the Ag_2_Se film fused at 150 ℃.


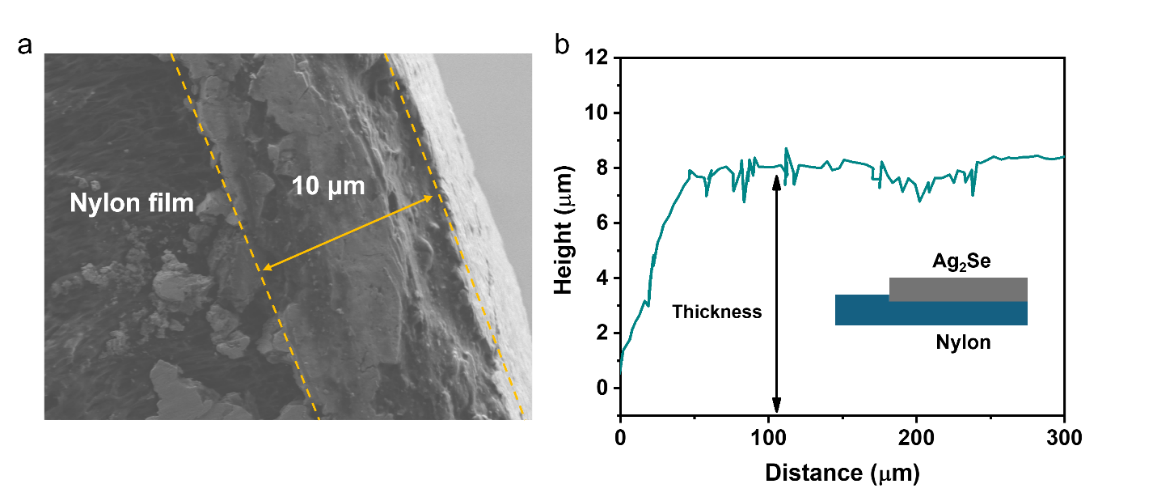


Figure S3: (a) The SEM image of the cross section of Ag_2_Se films. (b) Profiler measurement of the Ag_2_Se film on Nylon.


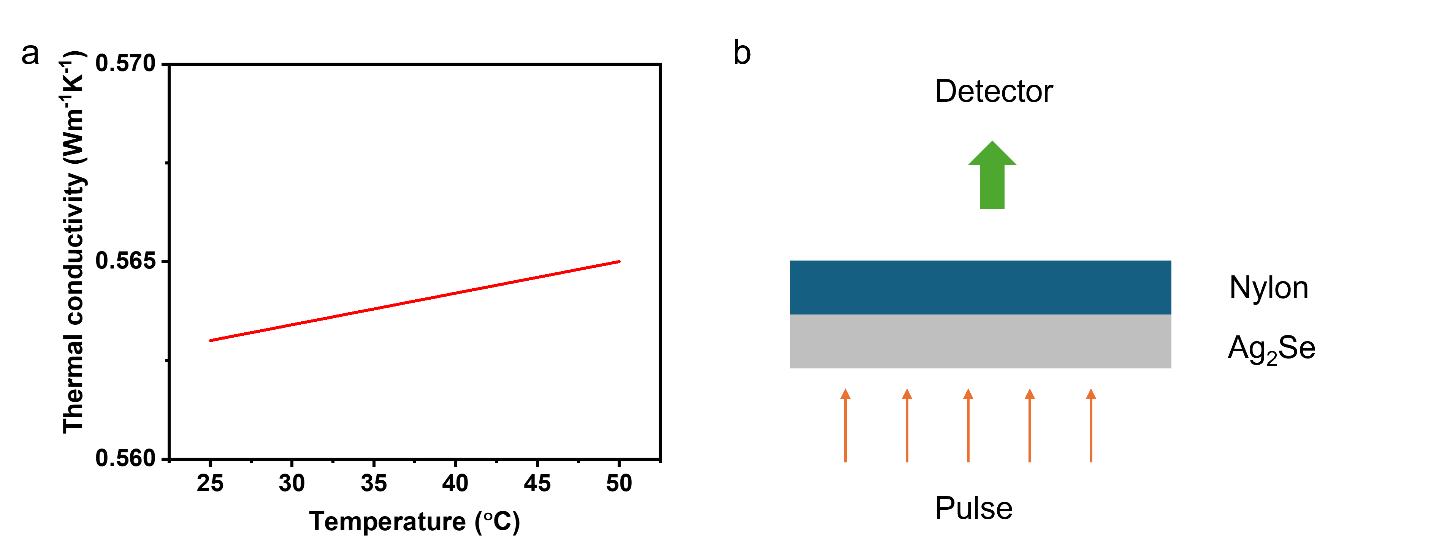


Figure S4: (a) Thermal conductivity with respect to temperature of Ag_2_Se and Nylon paper as a whole homogeneous material. (b) Laser flash (LFA) measurement scheme of Ag_2_Se on Nylon film.


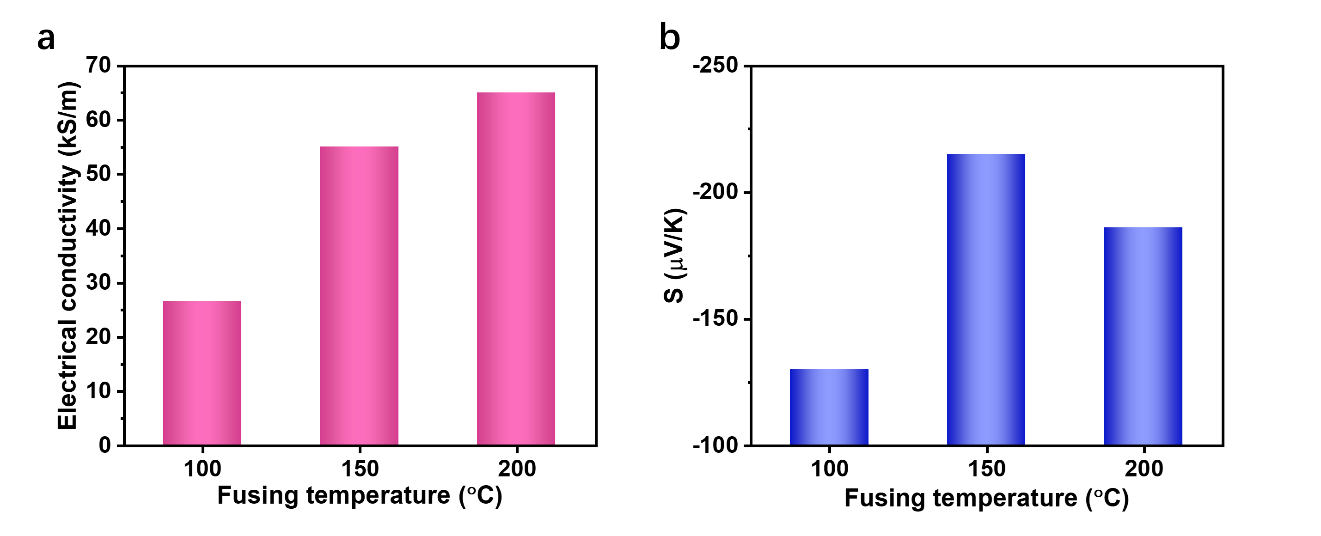


Figure S5: Comparison of (a) electrical conductivity and (b) Seebeck coefficient of Ag_2_Se films fused with different temperatures.


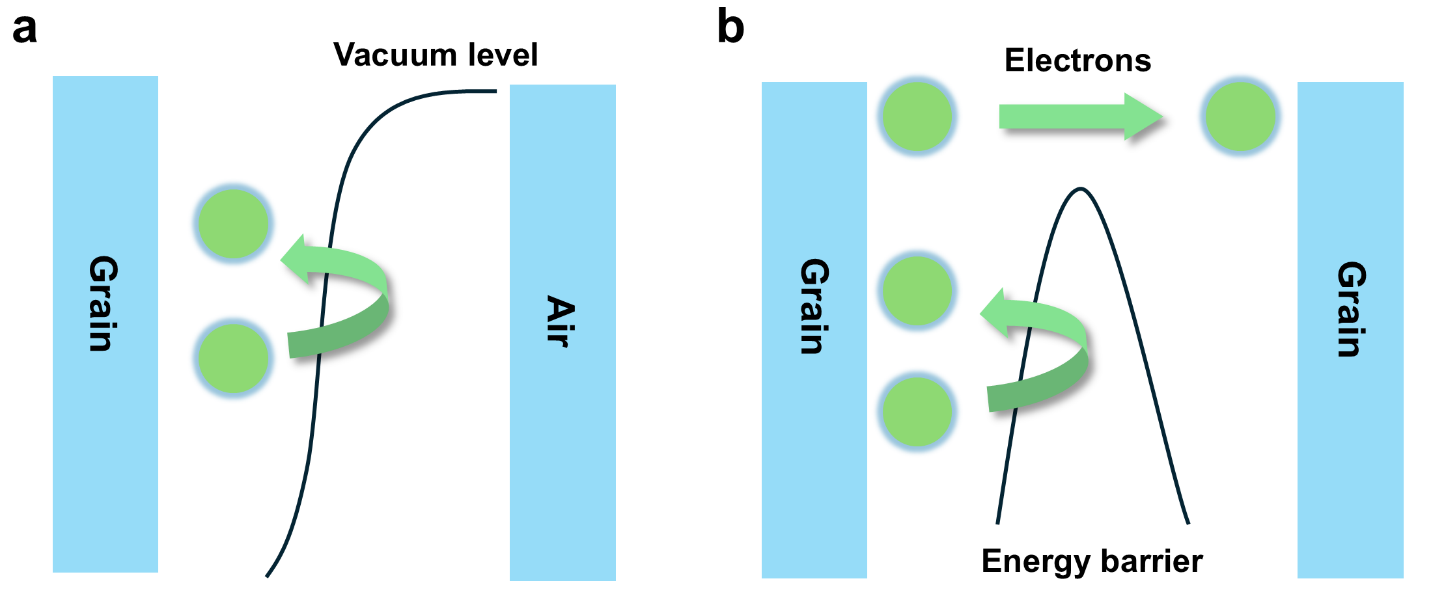


Figure S6: (a) The transportation of electrons between grains and air which does not induce energy filtering effect. (b) The transportation of electrons between grains and grains which does induce energy filtering effect.

**
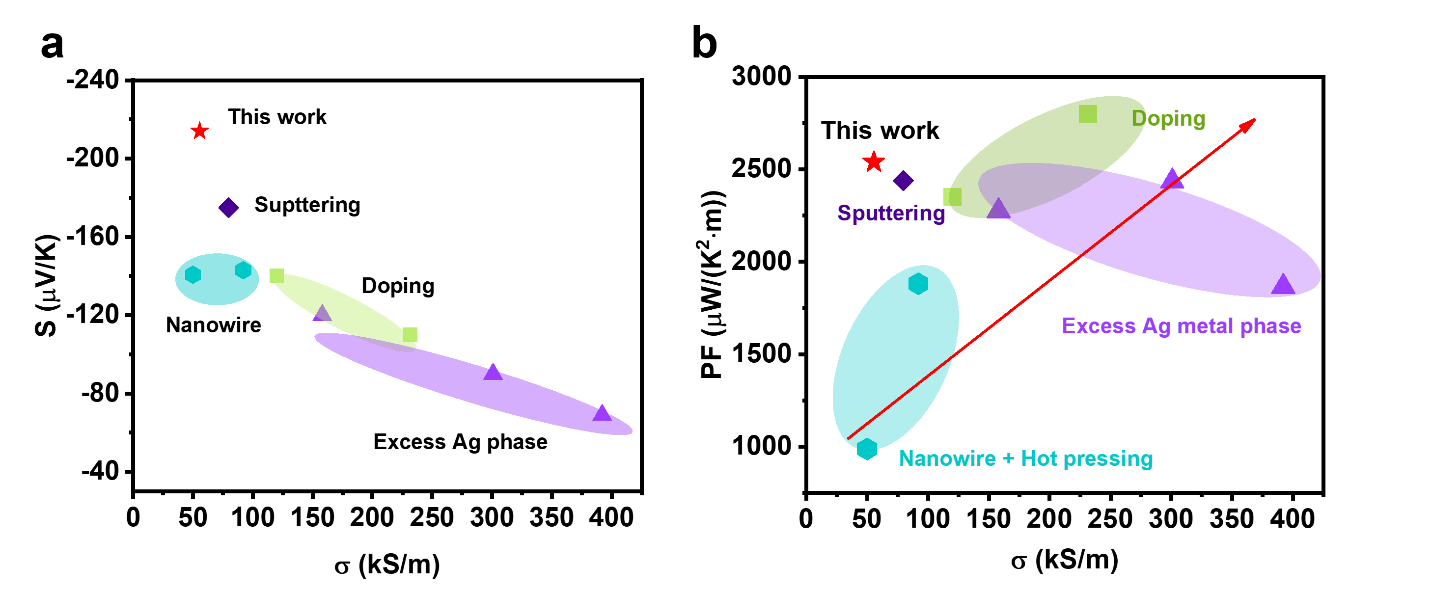
**

Figure S7: The comparison of (a) Seebeck coefficient vs electrical conductivity and (b) power factor vs electrical conductivity of our work with reference works.

**
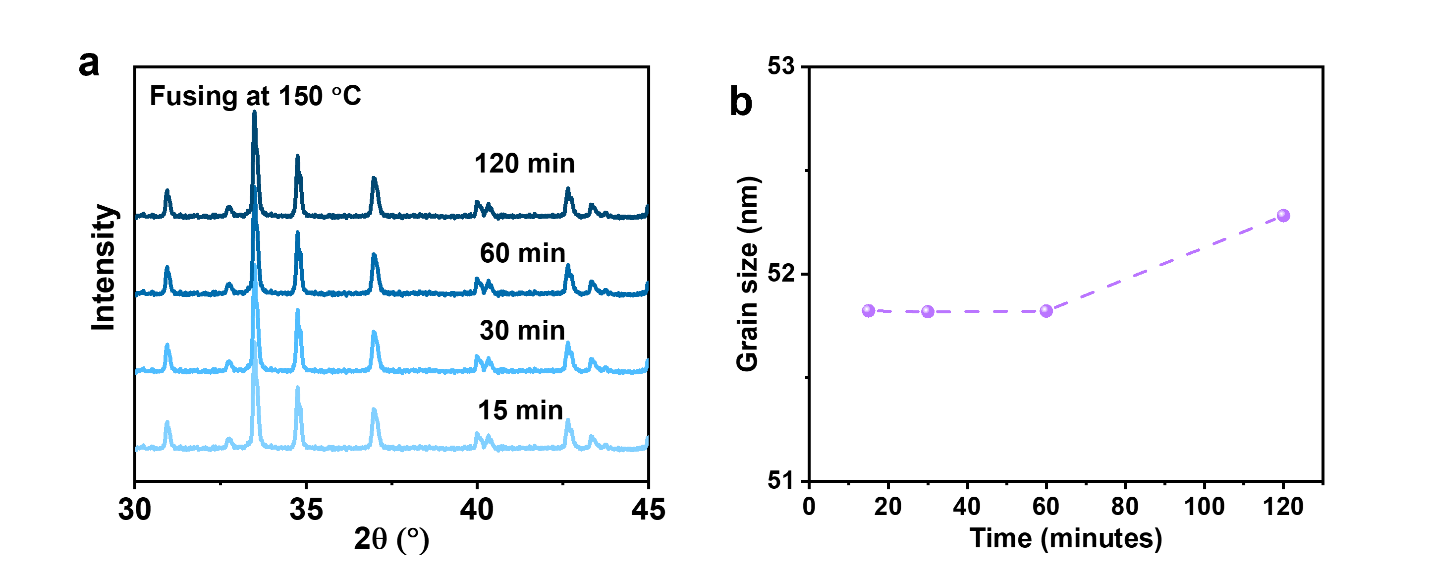
**

Figure S8: (a) X-ray diffraction (XRD) patterns of fused Ag_2_Se films at 150 ℃ with different fusing time and (b) corresponding gain sizes calculated from Scherrer equation.


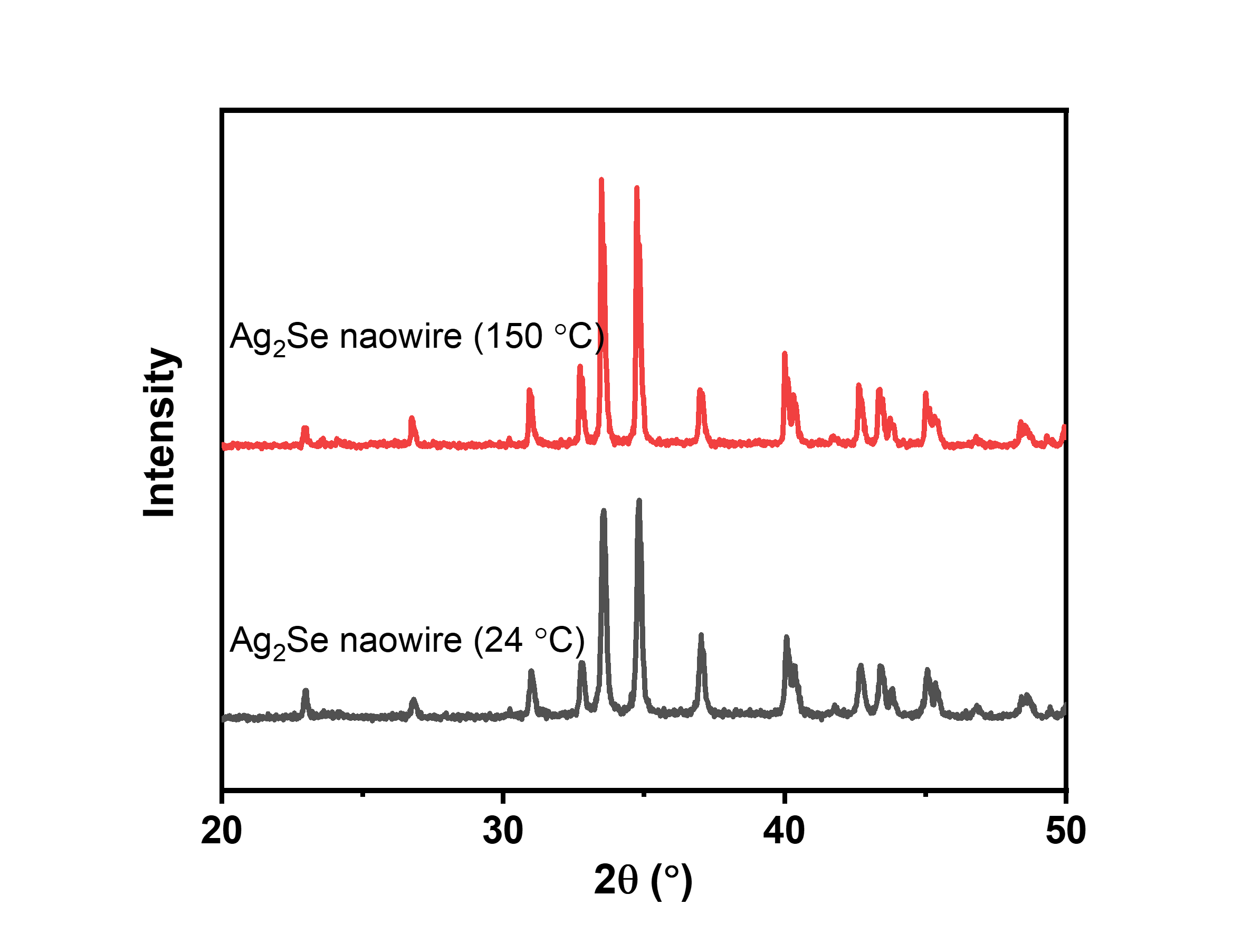


Figure S9: Comparison of XRD patterns of Ag_2_Se nanowire films fusing at room temperature and 150 ℃.


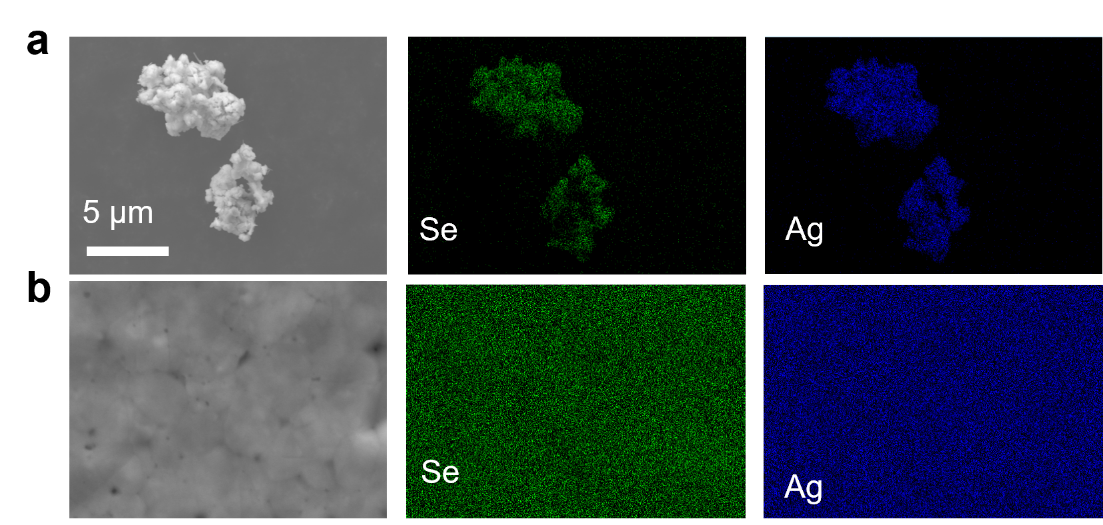


Figure S10: The energy-dispersive X-ray spectroscopy (EDS) mapping for (a) Ag_2_Se nanoparticles and (b) fused Ag_2_Se film (150 ℃).


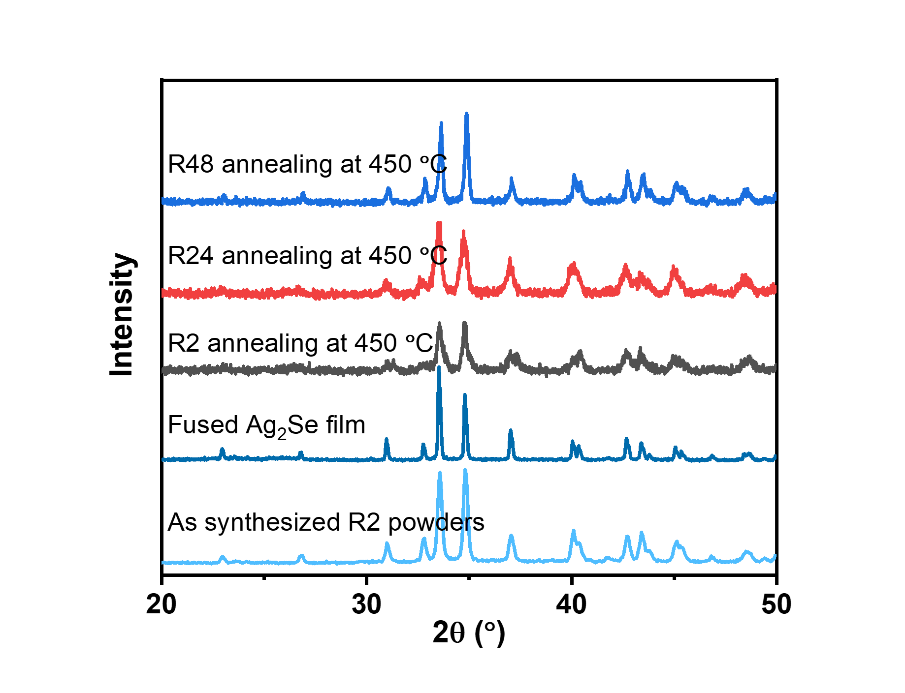


Figure S11: XRD patterns of R2 powders, Ag_2_Se film as well as annealed R2, R24 and R48 powders.


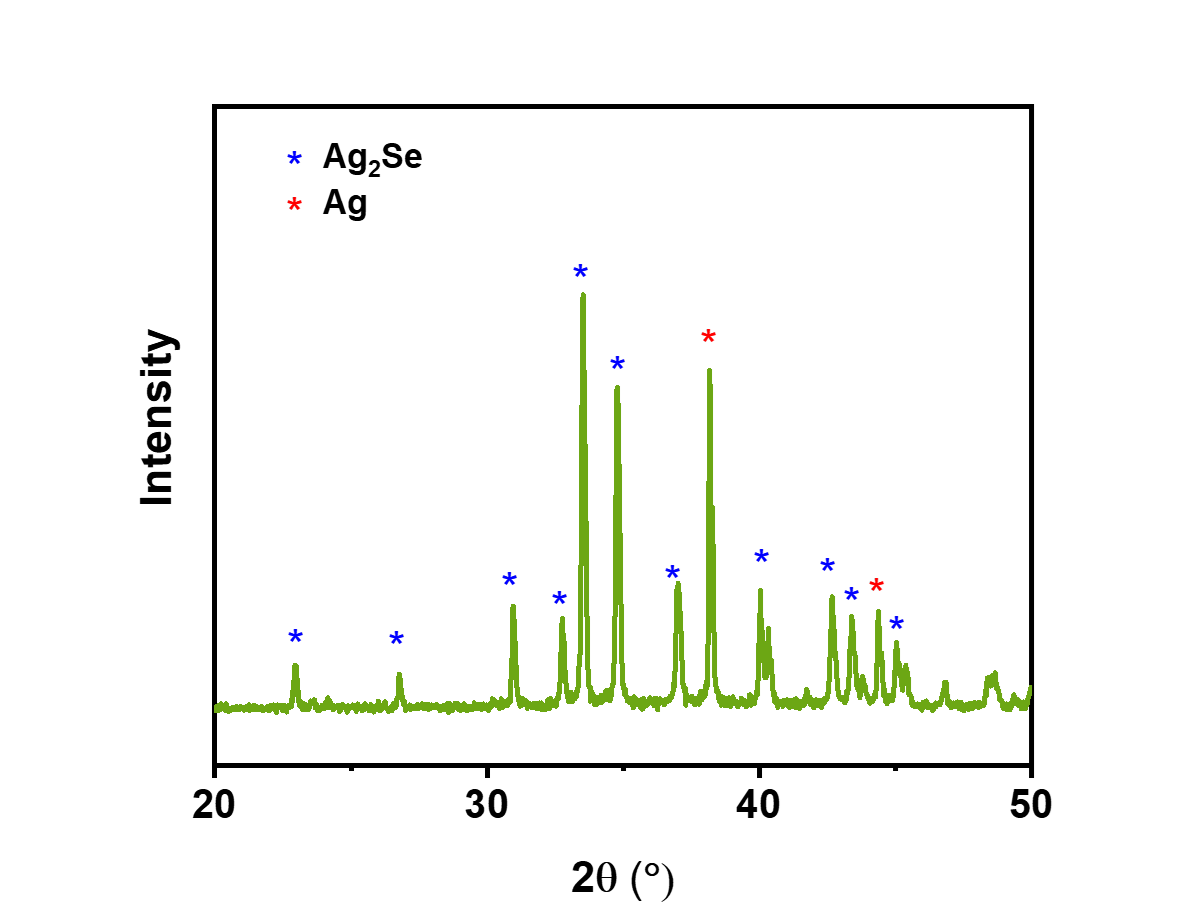


Figure S12: The XRD pattern of the Ag_2_Se film where strong reducing agent is employed during the synthesis of Ag_2_Se nanoparticles.


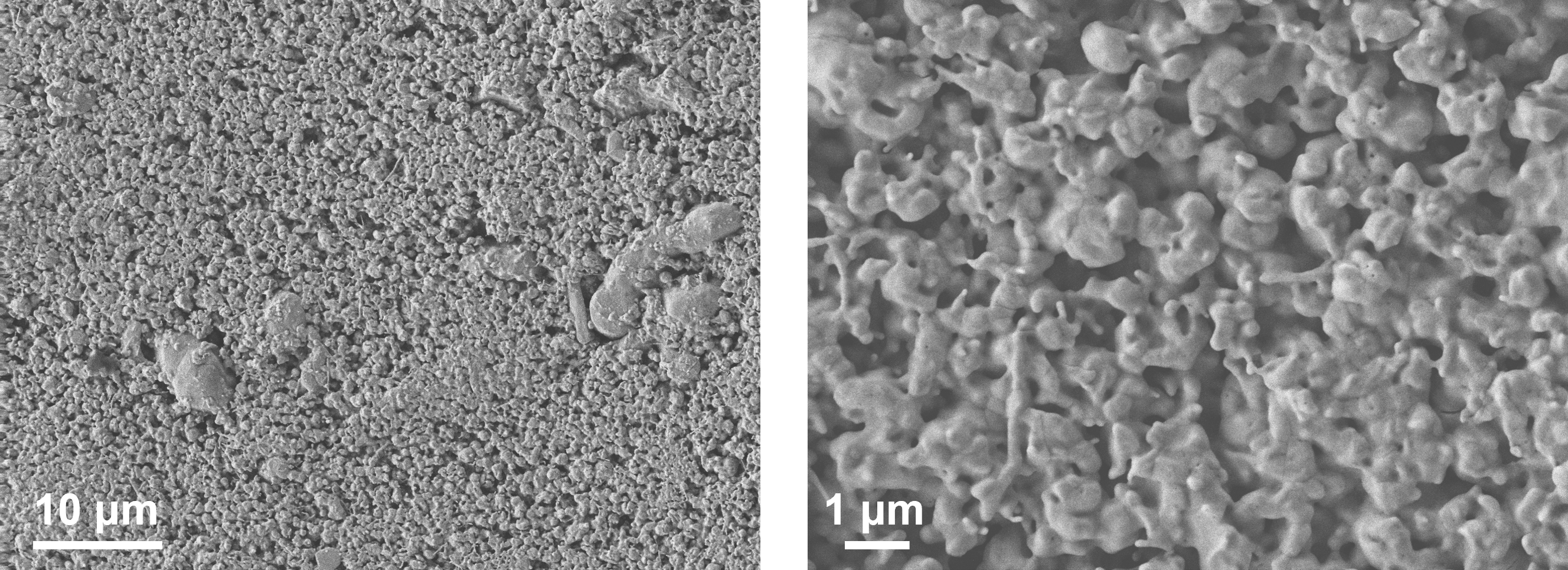


Figure S13: SEM images with different magnifications of the surface of Ag_2_Se film where strong reducing agent is employed during the synthesis of Ag_2_Se nanoparticles.

Table S1: Room temperature Seebeck coefficient and electrical conductivity reproduction from three different batches.

|  | Batch 1 | Batch 2 | Batch 3 |
| --- | --- | --- | --- |
| Seebeck coefficient (μV/K) | 214 | 198 | 205 |
| Electrical conductivity(kS/m) | 55 | 52 | 54 |

Table S2: The content of elements in fused Ag_2_Se film with respect to fusing temperatures (EDS in SEM)

| Fusing temperature (℃) | 24* | 100 | 150 | 200 |
| --- | --- | --- | --- | --- |
| C | 33.76 | 31.3 | 29.69 | 33.71 |
| Se | 21.75 | 22.06 | 22.76 | 20.75 |
| Ag | 44.29 | 46.64 | 47.59 | 45.53 |

*Where fusing at 24 ℃ means no fusing which are as-synthesized Ag_2_Se nanoparticles; and the unit for composition is atomic weight (%).

Table S3: The content of elements in 150 ℃ fused Ag_2_Se film (EDS from TEM)

|  | Ag_2_Se |
| --- | --- |
| C | 1.48 |
| O | 0.56 |
| Se | 30.46 |
| Ag | 67.50 |

The unit is atomic weight (%).

Table S4: The content of elements in fused Ag_2_Se film with respect to the reaction time of Ag_2_Se nanoparticles (EDS in SEM)

| Reaction time (hour) | 2 | 24 | 48 |
| --- | --- | --- | --- |
| C | 34.25 | 28.69 | 28.52 |
| Se | 21.99 | 22.76 | 22.69 |
| Ag | 43.76 | 46.56 | 48.79 |

The unit is atomic weight (%).

Table S5: The data of Ag/Se ratio, S and σ under different experimental conditions

| Sample | Ag/Se | S | σ | Changed conditions |
| --- | --- | --- | --- | --- |
| 1 | 1.99 | 138 | 27 | Reaction 2 hours |
| 2 | 1.97 | 151 | 36 | Reaction 24 hours |
| 3 | 2.02 | 200 | 52 | Se dispersion sonicates 2hours, reaction 48 hours |
| 4 | 2.05 | 152 | 39 | Reaction 24 hours L-Ascorbic acid: SeO_2_=2:1 |
| 5 | 2.15 | 198 | 51 | Reaction 48 hours, L-Ascorbic acid: SeO_2_=2:1 |
| 6 | 2.10 | 215 | 55 | Reaction 48 hours |
| 7 | 2.35 | 204 | 54 | AgNO_3_ solution reaction for 24 hours |
| 8 | 2.38 | 214 | 50 | AgNO_3_ solution reaction for 6 hours at 60 ˚C |
| 9 | 2.27 | 204 | 47 | Se nanowire, reaction for 12 hours |
| 10 | 2.62 | 136 | 140 | L-Ascorbic acid added in the reaction. |

The unit of S is μV/K and the unit of σ is kS/m. Except specially mentioned, other experimental conductions are the same as specified in the Methods section.
